# New approach methodologies for next-generation risk assessment of nanomaterials and nano-enabled products

**DOI:** 10.1186/s40580-026-00566-w

**Published:** 2026-07-22

**Authors:** Eunseo Lee, Aline Chary, Zayakhuu Gerelkhuu, Pamina Weber, Ishita Virmani, Beatrice A. Brugger, Iris Renata Sousa Ribeiro, Emilie Brun, Valentina Lacconi, Hyojoo Bang, Ik Hwan Kwon, Anna Pohl, Juyong Yoon, Shin Woong Kim, Anastasios Tassos Papadiamantis, Riju Roy Chowdhury, Dong-Youn Hwang, Lara Lamon, Melanie-Jasmin Ort, Martin Paparella, Ekaterina A. Sosnina, Angela Serra, Jack Morikka, Lorenzo Campini, Katharina Koch, Antreas Afantitis, Dario Greco, Iseult Lynch, Kristie Sullivan, Seokjoo Yoon, Tae Geol Lee, June-Woo Park, Diego Stéfani Teodoro Martinez, Vivekkumar P. Dadhania, Luisa Campagnolo, Winfried Neuhaus, Steffi Friedrichs, Tina Buerki-Thurnherr, Tae Hyun Yoon, Tommaso Serchi

**Affiliations:** 1 Department of Chemistry, Hanyang University, Seoul, 04763 Republic of Korea; 2 Environmental Health group - SUSTAIN Unit, Luxembourg Institute of Science and Technology, L4422 Belvaux, Esch-sur-Alzette, Luxembourg; 3 Research Institute for Convergence of Basic Science, Hanyang University, Seoul, 04763 Republic of Korea; 4 Institute of Medical Biochemistry, Medical University Innsbruck, 6020 Innsbruck, Austria; 5 Nanomaterials in Health Lab, Swiss Federal Laboratories for Materials Science and Technology (Empa), 9014 St. Gallen, Switzerland; 6 Brazilian Nanotechnology National Laboratory (LNNano), Brazilian Center for Research in Energy and Materials (CNPEM), Campinas, São Paulo Brazil; 7 School of Geography, Earth and Environmental Sciences, University of Birmingham, Birmingham, B15 2TT UK; 8 Department of Biomedicine and Prevention, University of Rome Tor Vergata, 00133 Rome, Italy; 9 Nanobio Measurement Group, Division of Biomedical Metrology, Korea Research Institute of Standards and Science, Daejeon, 34113 Republic of Korea; 10 AcumenIST SRL, 1040 Brussels, Belgium; 11 Korea Institute of Science and Technology (KIST) Europe Forschungsgesellschaft mbH, Campus E7.1, 66123 Saarbrücken, Germany; 12 Center for Ecotoxicology and Environmental Future Research, Korea Institute of Toxicology (KIT), Jinju, 52834 Republic of Korea; 13 Department of Cheminformatics, NovaMechanics Ltd., 1070 Nicosia, Cyprus; 14 Entelos Institute Ltd., 6059 Larnaca, Cyprus; 15 IUF – Leibniz Research Institute for Environmental Medicine, 40225 Düsseldorf, Germany; 16 Organoidsciences, Ltd., Osong, Chungbuk 28160 Republic of Korea; 17 ESQlabs GmbH, 26683 Saterland, Germany; 18 Finnish Hub for Development and Validation of Integrated Approaches (FHAIVE), Faculty of Medicine and Health Technology, Tampere University, 33100 Tampere, Finland; 19 DNTOX GmbH, 40223 Düsseldorf, Germany; 20 Department of Pharmacy, Frederick University, 1036 Nicosia, Cyprus; 21 Institute for In Vitro Sciences, Inc. (IIVS), Gaithersburg, MD 20878 USA; 22 Center for Biomimetic Research, Korea Institute of Toxicology (KIT), Daejeon, 34114 Republic of Korea; 23 SciQra LLC, Old Bridge, NJ 08857 USA; 24 Competence Unit Molecular Diagnostics, AIT Austrian Institute of Technology GmbH, 1210 Vienna, Austria; 25 Faculty of Medicine and Dentistry, Danube Private University, 3500 Krems, Austria; 26 Institute of Next Generation Material Design, Hanyang University, Seoul, 04763 Republic of Korea; 27 Yoon Idea Lab. Co., Ltd., Seoul, 04763 Republic of Korea

**Keywords:** New approach methodologies (NAMs), Nanotechnology, Nanomaterials, Next-generation risk assessment (NGRA), Regulatory acceptance, Safe and sustainable by design (SSbD)

## Abstract

New Approach Methodologies (NAMs) are increasingly promoted for animal-free nanosafety assessment, yet their regulatory use remains constrained by fragmented regulatory criteria and nanomaterial-specific testing complexities. This review comprehensively analyzes the convergence of advanced cell biology, nanotechnology, and information technology in reshaping safety evaluations, with a focus on regulatory translation across global frameworks. We first summarize enabling technologies, including advanced in vitro and ex vivo systems, organoids, microphysiological systems, high-dimensional single-cell multi-omics and label-free hyperspectral imaging, AutoML, generative AI, physiologically based kinetic modeling, and in vitro-to-in vivo extrapolation. We then analyze the regulatory landscape, emphasizing OECD harmonization efforts, the Mutual Acceptance of Data system, and regional developments across Europe, Africa, the Americas, and Asia–Pacific. A central theme is that regulatory acceptance of NAMs for nanomaterials requires not only biological relevance, reproducibility, and context-of-use definition, but also robust physicochemical characterization, exposure control, dosimetry, and data interoperability. Industrial implementation in pharmaceuticals, cosmetics, agrochemicals, antimicrobials, and environmental protection is discussed as a measure of regulatory readiness and scalability. Finally, we outline future directions involving Smart NAMs, digital twins, and Safe and Sustainable by Design (SSbD) frameworks. By integrating technological, regulatory, and implementation perspectives, this review identifies pathways toward harmonized, mechanistic, and human- or target species-relevant next-generation risk assessment (NGRA) for nanotechnology.

**Graphical abstract:**

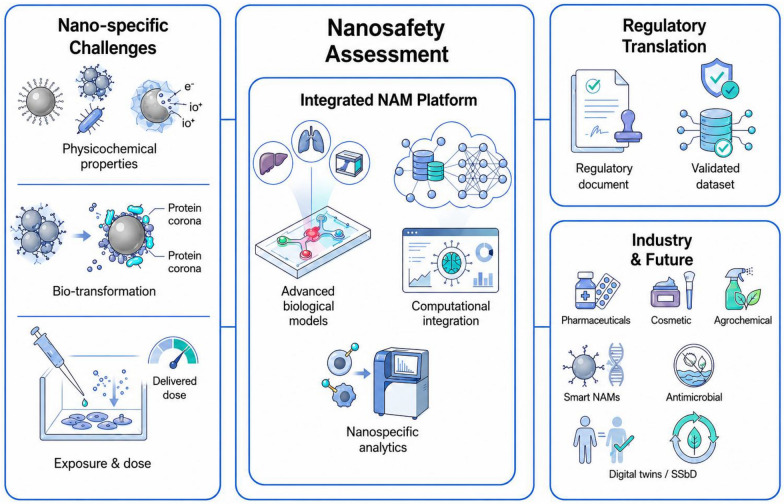

## Introduction

The traditional paradigm of toxicological safety assessment, which has relied heavily on vertebrate animal testing for nearly a century, is undergoing a fundamental transformation driven by a convergence of scientific necessity, ethical concerns, and economic pressures [1]. Historically, safety assessments relied on observing apical endpoints in animals—such as weight loss, organ pathology, or death—to predict human risks [2]. However, the predictive validity of these models has increasingly been questioned due to critical species-specific biological differences, which can contribute to high attrition rates in human clinical trials and the failure to identify critical human-specific adverse effects [3]. Moreover, animal tests have not traditionally been systematically validated for replicability, and recent reviews have begun to shed light on this considerable uncertainty [4].

To address these limitations, New Approach Methodologies (NAMs) have emerged as a cornerstone of “Toxicology in the 21st Century” [1, 5]. This transition is inspired by the 3Rs principles (Replace, Reduce, Refine) introduced by Russell and Burch, who bridged natural science with philosophy via their famous quote: “If we are satisfied that an experiment is maximally humane, we can be quite sure it is the most scientifically valuable one we could perform” [6]. The development of NAMs has therefore been closely associated with efforts to generate mechanistic knowledge, improve reproducibility, and address limitations of conventional animal-based models.

Despite their increasing use, NAMs are not yet defined uniformly across regulatory agencies, funding bodies, and scientific communities [7, 8]. Definitions proposed by organizations such as the European Chemicals Agency (ECHA), European Food Safety Authority (EFSA), U.S. Environmental Protection Agency (EPA), U.S. Food and Drug Administration (FDA), U.S. National Institutes of Health (NIH), and others differ in scope, with some emphasizing non-animal methods, others focusing on new regulatory decision-making strategies, and others referring more broadly to human- or target-species-relevant technologies [7–9]. This includes in vitro and ex vivo systems, in chemico assays, omics, in silico models, physiologically based kinetic (PBK) modeling, and in vitro-to-in vivo extrapolation (IVIVE) [9–11]. Integrated Approaches to Testing and Assessment (IATA) and Adverse Outcome Pathway (AOP)-informed frameworks are considered here as structures for organizing and integrating NAM-derived evidence, rather than as individual NAMs per se [12, 13].

Unlike traditional toxicology, largely descriptive and retrospective, NAMs emphasize mechanistic understanding of human biological perturbations and generate quantitative dose–response estimates. The Organisation for Economic Co-operation and Development (OECD) AOP framework supports the integration of NAMs by organizing assay outputs around biologically meaningful events, linking insights into Molecular Initiating Events (MIEs) to measurable Key Events (KEs) and to Adverse Outcomes (AOs) [12, 13].

Tox21 and ToxCast exemplify this transition [5, 14], while OECD Test Guidelines, IATA, and Defined Approaches have supported regulatory uptake [12, 15]. Policy momentum is accelerating: European Union (EU) Directive 2010/63/EU legislates the 3Rs, the European Commission is roadmapping a phase-out of animal testing [16], and the UK has published a national strategy for alternative methods [17, 18]. Similar initiatives are emerging across North America and Asia (Sect. 3). Consequently, NAMs have moved from supplementary tools toward essential components of next-generation risk assessment (NGRA) [12, 15, 17–23].

This regulatory momentum is matched by a parallel technological transformation. Early NAMs relied on simplified two-dimensional (2D) monocultures offering limited physiological relevance [24, 25]. Recent advances have been driven by the convergence of three complementary domains: nanotechnology, advanced cell biology, and information technology [9]. Progress in cell biology has yielded three-dimensional (3D) cultures, organoids, and microphysiological systems (MPS), including organ-on-chip models that more accurately recapitulate tissue architecture and dynamic exposure conditions [26–30]. Nanobiotechnology refines these microenvironments by modulating extracellular matrix properties, regulating exposure conditions, and improving structural control of advanced in vitro platforms [31, 32]. Information technology completes the triad: artificial intelligence (AI) and machine learning (ML) extract patterns from high-throughput datasets, support quantitative structure–activity relationship (QSAR) and nano-quantitative structure–activity relationship (nano-QSAR) modeling [33–36], and—integrated with PBK modeling and AOP frameworks—translate in vitro bioactivity into mechanistically interpretable, NGRA-relevant outputs [13, 19, 37].

**Fig. 1:**
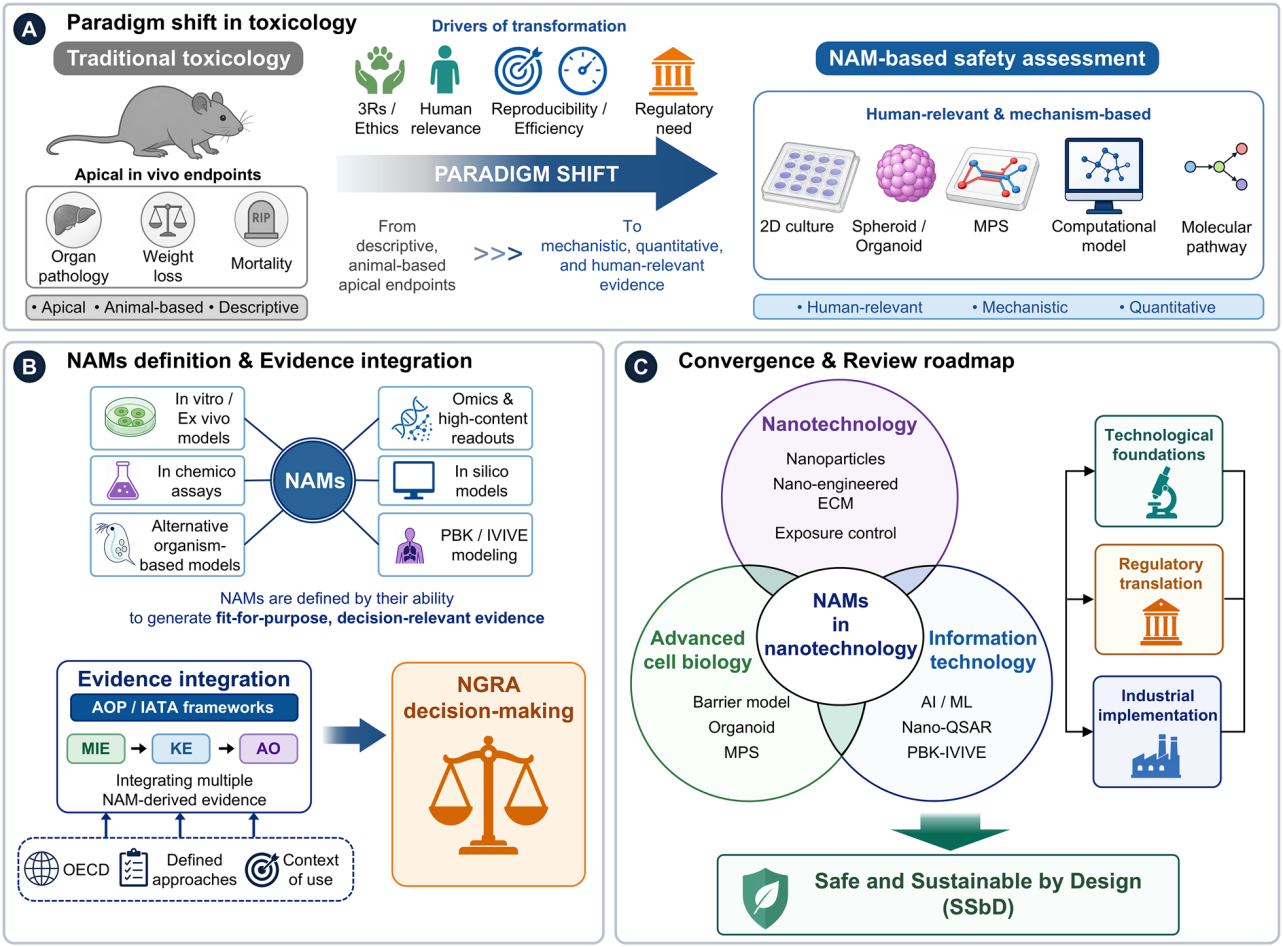
Conceptual framework and review roadmap for NAMs in nanotechnology

In this context, the present review provides a comprehensive, interdisciplinary analysis of NAMs in the context of nanotechnology, with particular emphasis on their technological foundations, regulatory translation, and industrial implementation. Rather than cataloguing all NAMs or restricting scope to nanotoxicology, it focuses on approaches used to assess nanomaterials and nanotechnology-based products, and on nanotechnology-informed tools that enhance advanced testing platforms—treating the convergence of nanotechnology, advanced in vitro systems, and AI as a key driver of next-generation predictive safety assessment. The review examines the technological pillars underpinning these approaches—2D/3D systems, organoids, MPS—and how nanobiotechnology enhances structural fidelity, dosimetry, and real-time monitoring. It explores in silico evolution from classical ML and nano-QSAR to automated machine learning (AutoML) and large language model (LLM)-assisted data extraction. Regulatory sections address global harmonization and regional divergences across OECD, Europe, the Americas, and Asia–Pacific, with particular attention to barriers—standardization, interlaboratory reproducibility, data quality—that perpetuate the "valley of death" between academic innovation and industrial adoption. Finally, the review situates NAMs within the broader paradigm of Safe and Sustainable by Design (SSbD) and digital transformation, outlining how integrating MPS, AI-driven analytics, and digital twin concepts may redefine safety assessment in the coming decade. By bridging technological innovation with regulatory and industrial realities, this review aims to provide a strategic perspective for accelerating the adoption of human-relevant, mechanistically informed, and context of use-driven safety assessment frameworks. Figure 1 provides the conceptual roadmap for the review. Panel A summarizes the shift from animal-based, apical endpoint testing to NAM-based, mechanism-informed evidence generation, while Panel B shows how diverse NAM evidence is integrated through AOP/IATA frameworks for NGRA decision-making. Panel C defines the central scope of the review: NAMs in nanotechnology arise at the intersection of nanotechnology, advanced cell biology, and information technology, and the review follows this convergence from technological foundations to regulatory translation, industrial implementation, and SSbD-oriented nanosafety assessment.

## Technological pillars of NAMs in nanotechnology: advanced biological platforms, novel analytical techniques, and in silico modeling

The transition from traditional toxicology toward predictive, human- and target species-relevant safety assessment relies heavily on three converging technological pillars: advanced biological test systems, novel analytical techniques, and computational modeling. For nanotechnology, this transition is particularly important because nanomaterial hazard is strongly influenced by particle identity, surface chemistry, agglomeration, dissolution, protein corona formation, sedimentation, and delivered dose. Therefore, NAMs for nanomaterials must combine biologically relevant models with robust physicochemical characterization, exposure control, and quantitative data interpretation.

Section 2 first summarizes the main in vitro NAM platforms used in nanotoxicology, ranging from 2D cell models to 3D spheroids and organoids, ex vivo systems, and MPS. It then discusses analytical technologies that enable high-content imaging, single-cell and spatial omics, and label-free particle tracking. Finally, it outlines in silico NAMs, including nano-QSAR models, machine learning, PBK modeling, IVIVE, AOP mapping, and emerging digital twin concepts.

### In vitro NAMs

In vitro NAMs provide controlled experimental systems for investigating nanomaterial–cell and nanomaterial–tissue interactions under defined exposure conditions. Their main advantage is the ability to isolate specific biological processes, quantify dose–response relationships, and generate mechanistic data that can support hazard identification, grouping, read-across, and NGRA. However, no single in vitro model can capture all relevant aspects of nanomaterial behavior. Model selection must therefore be driven by the regulatory or scientific context of use, the relevant exposure route, the biological question, and the level of complexity required [10, 19, 37].

For nanomaterials (NMs), the interpretation of in vitro data requires particular attention to dosimetry. Nominal concentrations often poorly reflect the actual cellular dose because nanomaterials may agglomerate, sediment, bind to proteins, interact with culture media, or interfere with assay readouts [37, 38]. Therefore, in vitro NAMs for nanotoxicology should ideally combine biological endpoints with characterization of administered dose, delivered dose, particle transformation, and potential assay interference.

**Fig. 2:**
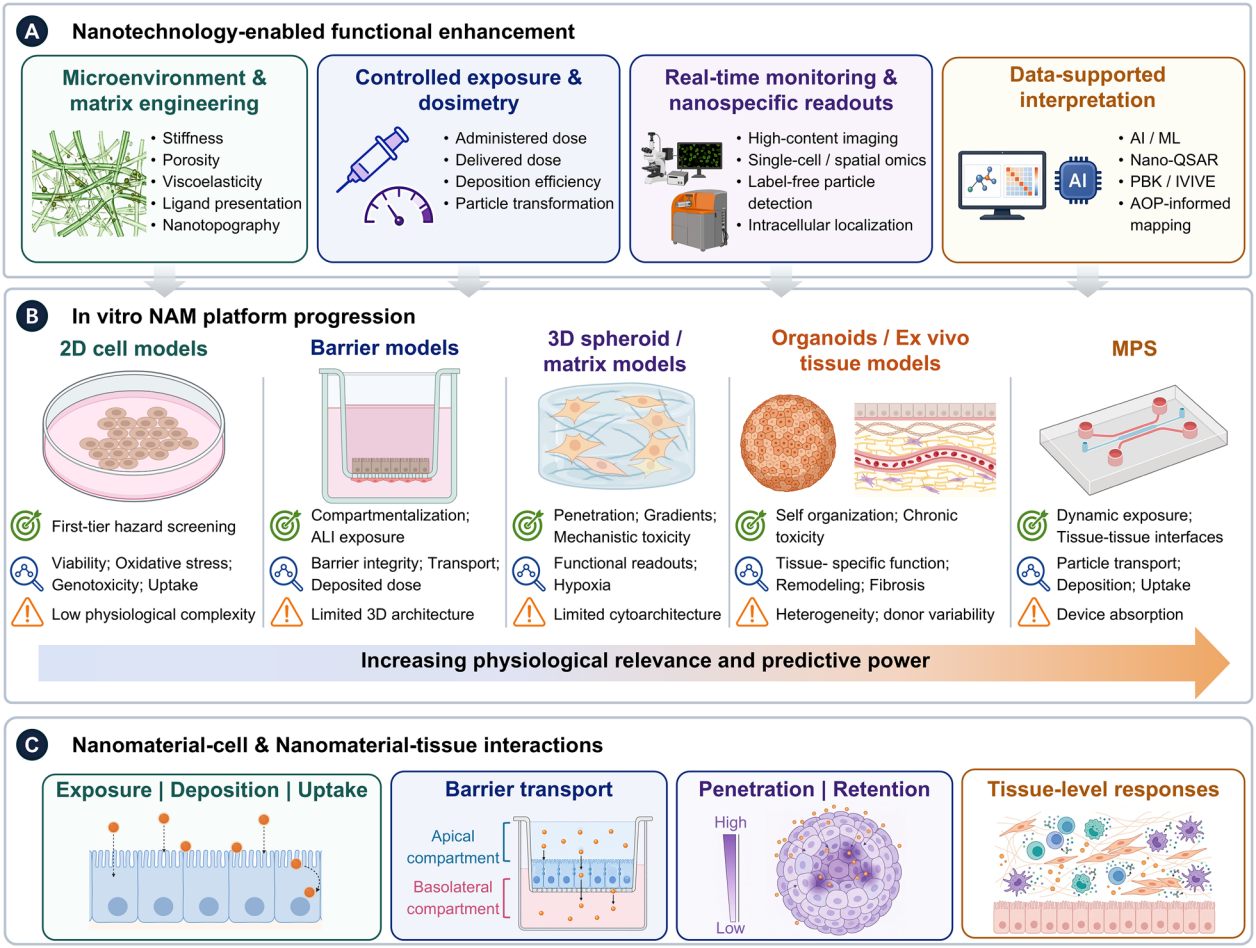
Evolution of in vitro NAM platforms toward physiologically relevant and predictive nanosafety assessment

Figure 2 provides a roadmap for the in vitro NAM platforms discussed in this section. Panel A summarizes nanotechnology-enabled platform enhancements, including matrix engineering, exposure control, real-time monitoring, nanospecific readouts, and data-supported interpretation. Panel B places 2D models, barrier systems, 3D spheroid or matrix models, organoids and ex vivo models, and MPS along a continuum of increasing physiological relevance and predictive power. Panel C then connects this progression to the nanomaterial–cell and nanomaterial–tissue interactions that these platforms are designed to assess, including exposure, uptake, barrier transport, penetration, retention, and tissue-level responses.

#### 2D, barrier, and 3D spheroid in vitro models

2D cell-based systems remain the most widely used in vitro NAM platforms because they are simple, cost-effective, reproducible, and compatible with automated high-throughput workflows [24, 25, 39]. They are particularly useful for first-tier nanomaterial hazard screening, dose–response assessment, and mechanistic studies. In nanotoxicology, 2D models are commonly used to compare materials with different physicochemical properties, including size, surface charge, coating, shape, crystallinity, dissolution behavior, and chemical composition [25]. Typical endpoints include cell viability, membrane damage, oxidative stress, inflammatory mediator release, genotoxicity, mitochondrial dysfunction, and cellular uptake. These assays can provide early information on material reactivity and mode of action, especially when combined with physicochemical characterization and appropriate controls for assay interference.

2D systems are also relevant to ecotoxicology, where fish cell lines and other cell models from environmentally relevant species can help reduce reliance on vertebrate testing [40, 41]. A key example is the RTgill-W1 fish cell line assay, adopted as OECD Test Guideline (TG) 249 for acute fish toxicity assessment [42]. Such models are useful for screening aquatic toxicity and for investigating cellular responses to nanomaterials under controlled conditions.

Despite these advantages, 2D cultures present important limitations. They lack tissue architecture, extracellular matrix (ECM), physiological gradients, and apical–basolateral polarity [25, 43], which can lead to biological responses that differ from those observed in more complex tissue models. In nanomaterial studies, including those using environmental cell models, interpretation is further complicated by particle sedimentation, agglomeration, dissolution, and differences between nominal and delivered dose [37].

To address some of these limitations, insert-based culture systems provide an important intermediate step toward increased physiological relevance, particularly for biological barrier studies. Cells are cultured on porous, permeable membranes, thereby allowing apical–basolateral compartmentalization, barrier formation, and independent sampling from different compartments [44, 45]. These systems are widely used to model biological barriers such as the lung, skin, intestine, blood–brain barrier (BBB), and placenta [44–46]. For nanomaterials, they enable the assessment of permeability, translocation, barrier integrity, apical versus basolateral responses, and vectorial transport.

Air–liquid interface (ALI) models are particularly important for inhalation toxicology because they allow direct aerosol or particle deposition onto the apical surface [47]. This is more physiologically relevant than submerged exposure of inhaled nanomaterials, provided that deposition efficiency, delivered dose, aerosol properties, and particle transformation are well characterized [47].

3D spheroids and engineered matrix-based (e.g. hydro- or microgel) systems further increase biological relevance by introducing multicellular organization, cell–cell communication, ECM interactions, and oxygen or nutrient gradients, resulting in cellular behaviors that more closely resemble those observed in native tissues [25, 43, 48–53]. These features make spheroid and matrix-based systems useful not only for studying nanoparticle penetration, retention, and spatially heterogeneous responses, but also for assessing mechanistic and functional toxicity in standardized multicellular settings [51, 54–56]. Moreover, the generation of 3D spheroids from human induced pluripotent stem cells (hiPSCs) enables application of robust quality control frameworks and the use of genome-engineered lines, ultimately increasing reproducibility and biological coverage [57–59]. Tumor spheroids, for example, have shown that nanoparticle penetration depends on size, matrix density, and cell packaging [60]. Smaller particles may diffuse deeper into spheroids, whereas larger or more strongly interacting particles often remain near the periphery [51, 60, 61].

Nanoengineered matrices and nanostructured scaffolds can further improve control over the microenvironment by tuning stiffness, porosity, viscoelasticity, ligand presentation, and nanotopography [31, 32, 62, 63]. In this context, nanomaterials have a dual role: they can be used as engineering tools to improve model architecture, while also serving as test materials requiring safety evaluation [31, 32, 60–66]. The main challenge is to balance improved biological relevance with reproducibility, scalability, and standardization [39, 67]. The main applications and limitations of 2D, insert-based, and 3D models are summarized in Table 1.

**Table 1:** Comparison of 2D, insert-based, and 3D in vitro models for nanotoxicology

Model	Main use	Nano-specific value	Key readouts	Key limitation
2D monolayers	Screening, mechanisms	Compare NM properties under controlled conditions	Viability, ROS, cytokines, genotoxicity, uptake	Low physiological relevance
Insert barriers	Transport, permeability	Apical/basolateral exposure and sampling	TEER, permeability, translocation	Limited 3D architecture
ALI models	Inhalation exposure	Direct aerosol/particle deposition	Deposited dose, inflammation, barrier effects	Aerosol delivery and deposited dose quantification
3D spheroids	Penetration, organ-specific, mechanistic toxicity	Models diffusion barriers and spatial responses	Penetration, uptake, hypoxia, mechanistic/functional/cell type-specific readouts	Limited 3D cytoarchitecture
Engineered matrices	Matrix-dependent effects	Tunable stiffness, porosity, nanotopography	Matrix interaction, morphology, matrix remodeling	Matrix reproducibility and standardization challenges

Taken together, 2D, insert-based, and 3D spheroid systems provide complementary levels of complexity for nanotoxicology, generating mechanistic toxicological information while maintaining sufficient throughput for screening approaches. Simple 2D models remain valuable for scalable screening and mechanistic comparison, while insert-based and ALI models improve exposure relevance for biological barriers. Multicellular spheroids and engineered 3D matrices add tissue-like organization and spatial gradients, while retaining compatibility with standardized screening workflows [60]. However, increasing complexity must be matched by improved standardization, exposure characterization, and quality control to ensure regulatory and industrial usability [10, 39, 67].

#### Organoid and ex vivo models

Organoids provide a higher level of biological complexity than conventional 2D or spheroid systems. Organoids are self-organizing, 3D structures derived from stem cells, progenitor cells, or tumor-derived cells. They can reproduce key aspects of organ architecture, cellular heterogeneity, and tissue-specific function, making them valuable for studying organ-specific toxicity, chronic responses, metabolism-dependent effects, tissue remodeling, and developmental toxicity [27, 68–70].

These features make organoid-based test systems highly promising for NM hazard characterization across multiple target organs when applied within defined NAM workflows [27, 68]. Reviews of organoids in nanotoxicology consistently emphasize these advantages, while also noting that transport, penetration, protein corona formation, and cell-type-specific responses can differ markedly from simpler in vitro models [27].

Although simplified 2D monolayers remain useful for first-tier screening and mechanistic comparison, they cannot fully capture the diffusion barriers, ECM organization, multicellular architecture, or cell-type-specific susceptibility that shape nanoparticle penetration, local retention, and spatially heterogeneous responses. Static 2D exposure can also promote agglomeration or sedimentation, causing nominal administered concentrations to diverge from delivered cellular doses [37, 38]. Organoid architecture therefore adds more than biological complexity: it provides a tissue-like environment for evaluating nanomaterial transport, local retention, and biological responses. Accordingly, organoids can bridge material characterization and biological response by capturing tissue-context-dependent changes, including, where relevant, protein-corona evolution and particle transformation. These changes can then be interpreted alongside delivered dose and downstream biological responses [27, 60, 61, 71].

Different organoid systems provide complementary opportunities for organ-specific nanosafety assessment. Liver organoids can support the assessment of metabolism-associated toxicity [69], pulmonary organoids can model fibrotic and remodeling responses to inhaled materials [72], and cerebral organoids may help identify neurodevelopmental effects of nanomaterials [73]. However, organoids often lack mature vascularization, immune components, mechanical cues, and fully developed barrier functions [74].

Emerging assembloid approaches, which combine different organoids or organoids with specialized cell types, can further expand organoid-based test systems by enabling the study of cell–cell and tissue–tissue interactions that are difficult to capture in single-organoid systems [70, 75]. Although their application to NM safety assessment remains at an early stage, assembloids may become useful for modeling tumor–stroma interactions, neuroimmune or neurovascular crosstalk, and barrier–tissue communication [70, 75].

Ex vivo models, such as precision-cut tissue slices, placental explants, and tissue biopsies, preserve native tissue architecture, ECM, and resident cell populations [76–78]. They are useful for studying tissue-level inflammation, fibrosis, mediator release, and nanoparticle penetration in a native biological context [76–78]. Their main limitations are restricted tissue availability, donor variability, short culture windows, possible preparation-induced injury, and exposure conditions that may not fully reproduce physiological exposure routes [79]. The key features of organoid and ex vivo models are summarized in Table 2.

**Table 2:** Comparison of organoid and ex vivo platforms for nanotoxicology

Model	Main use	Nano-specific value	Key limitation
Liver organoids	Organ-specific metabolism and longer-term function	Metabolism-dependent toxicity, persistence, secondary responses	Variable maturity; limited vascularization
Lung organoids	Pulmonary epithelial organization and tissue remodeling	Fibrosis, remodeling, inflammatory responses to inhaled NMs	Limited immune/vascular components; exposure-route challenges
Cerebral organoids	Human-relevant neuro(developmental) biology	Neurotoxicity, neurodegeneration	Lack of mature BBB and vascularization; reproducibility
Tumor organoids	Patient- or tumor-specific architecture	Nanomedicine efficacy, penetration, retention, tumor-specific toxicity	Heterogeneity and matrix-dependent access
Assembloids	Interactions between organoids or specialized cell types	Neurovascular, neuroimmune, tumor–stroma, barrier–tissue crosstalk	Emerging technology; limited standardization
Precision-cut tissue slices	Preserved native architecture and resident cells	Tissue-level toxicity, fibrosis, inflammation, particle penetration	Short viability window, slicing injury, donor variability
Placental explants	Native placental barrier and secretome	Placental transfer, secretome-mediated downstream effects	Donor variability, limited culture duration

#### Organ-on-chip and MPS

Building on organoid and ex vivo models, MPS, including organ-on-chip platforms, integrate biological models with microengineering, controlled perfusion, and dynamic exposure conditions. Compared with static cultures, these systems can reproduce key physiological features such as fluid flow, shear stress, oxygen and nutrient gradients, mechanical stimulation, and tissue–tissue interfaces [28, 29, 80].

For nanomaterial testing, fluidics in MPS helps address a central uncertainty in nanotoxicology: the mismatch between nominal and delivered dose. Under static exposure, particles may agglomerate, settle, or deposit onto cells in material- and medium-dependent ways that may not reflect physiological exposure [37]. By controlling flow, residence time, transport kinetics, and shear stress, perfused MPS can improve delivered-dose interpretation and support assessment of particle transport, uptake, barrier translocation, and dynamic tissue responses [30, 81, 82].

Single-organ chips have been developed for liver, kidney, lung, brain, placenta, intestine, and other tissues [28, 29, 80, 83]. Barrier-on-chip models are particularly relevant for nanotoxicology because they allow independent perfusion of apical and basolateral compartments, enabling the study of translocation across interfaces such as the air–blood barrier, intestinal barrier, blood–brain barrier, and placenta [29, 30]. Multi-organ systems extend this further by linking organ modules to investigate metabolism-dependent toxicity, secondary mediator effects, and downstream organ responses [83, 84].

Despite their potential, MPS platforms face important implementation challenges. These include device material absorption, especially with polydimethylsiloxane (PDMS); limited throughput; inter-platform variability; scaling of organ compartments; common medium compatibility; and the need for standardized operating procedures [85–88]. For nanomaterial studies, these challenges also include possible particle adsorption or deposition within tubing, membranes, or chip materials, as well as the need to verify particle recovery, dispersion stability, and exposure profiles under flow [30, 81, 82, 85–88]. For regulatory use, their value depends on clearly defined contexts of use, technical qualification, reproducibility, and integration with quantitative modeling [10, 67, 89]. The main MPS formats relevant to nanotoxicology are summarized in Table 3.

**Table 3:** Comparison of MPS and organ-on-chip platforms for nanotoxicology

Model	Key feature	Nano-specific value	Main challenge
Single-organ chip	Perfused tissue model with controlled flow	Dynamic uptake, chronic exposure, tissue-specific toxicity	Platform complexity and limited standardization
Barrier-on-chip	Two compartments separated by a biological barrier	Translocation across lung, gut, BBB, placenta, or skin barriers	Barrier validation and scaling
ALI lung-on-chip	Air-side exposure with perfused basal compartment	Inhalation-relevant aerosol or particle exposure	Aerosol delivery and deposited-dose quantification
Multi-organ chip	Interconnected organ modules	Indirect toxicity, metabolism-dependent effects, systemic signaling	Complex scaling and common medium formulation
High-throughput MPS	Parallelized culture and imaging	Screening of NM libraries, phenotypic profiling	Automation, image analysis, and data harmonization
Alternative-material chips	Thermoplastics, glass, low-absorption polymers	Improved dose accuracy compared with PDMS	Fabrication and scalability

### Novel analytical techniques for NAMs

Advanced analytical techniques are essential to NAM implementation because they generate high-dimensional, spatially resolved, and phenotypically informative datasets that reveal cellular heterogeneity, temporal dynamics, tissue organization, and nanomaterial–biological interactions. In nanomaterial applications, these platforms must also capture particle state, localization, and exposure-dependent transformation while accounting for readout interference, rather than treating these factors as secondary experimental artifacts. This section therefore discusses complementary analytical platforms, including single-cell and spatial omics, high-throughput and high-content phenotypic imaging, and label-free particle detection and localization. Together, these approaches connect nanomaterial-specific experimental readouts with mechanistic interpretation, supporting AOP-informed, data-driven risk assessment and regulatory decision-making [8, 10, 39].

#### Multi-omics profiling platforms at single-cell resolution

For NMs, high-resolution profiling is important because biological responses can vary across cell types, tissue regions, and local exposure microenvironments due to differences in particle uptake, intracellular localization, agglomeration state, surface transformation, and protein corona formation. These differences may arise from variation in particle uptake, intracellular localization, agglomeration state, surface transformation, and protein-corona formation, which can redefine the biological identity of NMs. Population-averaged readouts may therefore obscure rare responder populations, cell-type-specific susceptibility, or spatially restricted perturbations relevant to mechanistic hazard assessment. Even under the same nominal exposure condition, cellular NM association and downstream responses may differ among individual cells, making single-cell analysis important for interpreting heterogeneous NM effects [90–92].

Single-cell molecular profiling technologies, including single-cell RNA sequencing (scRNA-seq) and mass cytometry (CyTOF), enable the identification of rare subpopulations, transitional cellular states, and divergent response trajectories following NM exposure. In complex 3D models such as organoids and MPS, scRNA-seq can distinguish cell lineages, identify sensitive or resistant subpopulations, and map transitional phenotypes that would otherwise be masked in population-level measurements [93, 94]. Recent NM-focused scRNA-seq studies further support this point by resolving cell-type-specific transcriptional responses to nanomaterial exposure, including injury- and inflammation-related signatures that may be obscured in bulk-level analyses [95].

CyTOF complements transcriptomic profiling by using metal-tagged antibodies and time-of-flight mass spectrometry to quantify more than 40 cellular parameters or protein markers simultaneously. This enables high-dimensional proteomic profiling and is particularly compatible with inorganic NMs [96, 97]. Because CyTOF avoids optical interference and quenching common in fluorescence-based methods, it can be used to correlate signaling activation, including phosphorylated protein cascades, with cell-type-specific nanoparticle (NP) association or uptake. For metal-containing or metal-labeled NMs, CyTOF and related single-cell elemental approaches can further link cellular particle association with phenotypic, inflammatory, signaling, and cell-death markers in the same cell [98]. Primary and complementary studies collectively illustrate this value: AgNP exposure in human immune cells revealed subset-dependent particle association and stress-related responses; AuNP studies showed that protein corona formation can alter cellular association and uptake routes. Together, scRNA-seq and CyTOF provide complementary transcript- and protein-level views of NM-induced perturbations across heterogeneous cell populations [91, 99–102].

Spatially resolved technologies further extend these approaches by preserving the positional context of molecular and cellular responses within tissue-like NAM platforms. Spatial transcriptomics, spatial proteomics, multiplex immunofluorescence, and imaging mass cytometry can localize NM-induced perturbations to specific tissue regions, cell types, or microenvironmental niches, which is especially relevant for organoids, MPS, and barrier models where exposure gradients and particle localization shape outcomes. These approaches directly address where NM accumulation, inflammatory activation, stress adaptation, or tissue remodeling emerges within complex NAM platforms. High-content phenotypic imaging can complement these readouts and is discussed in the following section [103–106].

By integrating single-cell, spatial, and phenotypic data streams, NAM workflows can support linkage between MIEs, KEs, and AOs, provide datasets for computational models and digital twins, and advance AOP-anchored predictive toxicology. For regulatory use, these approaches require defined contexts of use, harmonized sample preparation and marker panels, standardized data-processing pipelines, transparent quality-control metrics, and biologically interpretable links to KEs or AOP-informed hypotheses [10, 107, 108].

#### High-throughput and high-content profiling platforms

To address the low throughput of early MPS designs, recent advances have focused on automated platforms capable of culturing hundreds of organoids simultaneously. Technologies such as microfluidic large-scale integration (mLSI) chips and micropillar arrays enable the parallel generation and culture of standardized 3D organoids or microtissues, reducing batch-to-batch variability and supporting high-throughput, high-content screening applications. Such high-throughput and high-content platforms can facilitate the industrial implementation of NAMs by enabling scalable, multiparametric screening of NM toxicity across relevant in vitro model systems [109, 110]. Practical implementations include high-throughput organo-on-pillar (High-TOP) and U-shaped pillar-strip–based 384-well platforms, which use automated spotting or arrayed pillar architectures to generate standardized 3D models for high-throughput image-based phenotypic analysis [109, 110].

The true potential of these high-throughput platforms is realized when integrated with advanced optical imaging techniques that capture functional dynamics non-invasively or with minimal perturbation. Complementary modalities, including high-content fluorescence imaging, confocal microscopy, light-sheet microscopy, Raman-based imaging, optical coherence tomography, and multiplexed live-cell imaging, can further provide information on morphology, viability, tissue architecture, barrier integrity, and stress-response phenotypes [65, 111, 112]. For instance, high-speed digital holographic microscopy can monitor real-time phase dynamics and heart-rate changes in aquatic bioassays, including *Daphnia magna* cardiotoxicity assessment [113]. Complementary automated approaches for *Daphnia* cardiac readout have been developed independently by other groups, ranging from high-speed videography with ImageJ-based quantification to deep-learning–based methods using U-Net/Mask R-CNN segmentation and DeepLabCut markerless tracking, with application to pesticide and chemical cardiotoxicity assessment [114–116]. Such imaging modalities are particularly valuable for NM toxicity assessment because they provide scalable functional and phenotypic readouts without perturbing the biological system. In an ecotoxicological extension of this concept, high-speed optical imaging coupled with Fourier-based frequency analysis has been used to monitor cardiac dynamics in *Daphnia magna* at population scale [117]. By achieving a throughput of up to 150 individuals per hour, this system enables a transition in toxicological paradigms from conventional mean-based values to statistically robust, probabilistic distribution analyses. The use of *Daphnia* heart rate as a sub-lethal endpoint for nanomaterial hazard has been independently corroborated across diverse nanomaterial classes, including fullerenes [118], metallic and metal-oxide nanoparticles such as TiO₂ and Ag [119, 120], graphene oxide [121], and polymeric nanoplastics [122], reinforcing the broader applicability of cardiac readouts for sub-lethal nanotoxicity assessment.

In the context of nanotoxicity, this high-throughput optical approach is essential for capturing the stochastic nature of NM–biological interactions. By analyzing the distribution of parameters such as heart rate variability across large populations, researchers can identify subtle toxicological signatures that are often obscured in small-scale studies. This shift from point estimates to distributional analyses is particularly significant when assessing NMs, whose biological effects are highly dependent on physicochemical properties such as size, surface chemistry, surface charge, agglomeration state, and delivered dose [123–127]. The rationale for distribution-based rather than mean-based interpretation is well established beyond the *Daphnia* model: across toxicology and pharmacology, population-averaged endpoints are recognized to obscure heterogeneous responders and sensitive subpopulations, thereby concealing divergent responding within group means [128]. Population-scale in vitro screening of genetically diverse human cell panels has been used specifically to quantify inter-individual toxicodynamic variability and to identify hyper-sensitive subpopulations that point estimates would miss [129]. Population-resolved cardiac readouts therefore align with a broader regulatory shift toward characterizing susceptible subpopulations in NGRA.

Furthermore, high-throughput optical datasets can provide multiparametric imaging features for predictive toxicological models, but their utility for early-toxicity detection and NM hazard-profile classification depends on sufficiently large, population-derived training datasets and external validation. Ultimately, integrating automated culture platforms with high-resolution, high-speed optical analysis can improve the throughput, reproducibility, and reliability of NM safety assessment.

#### Label-free dark-field hyperspectral imaging platforms

While the fluorescence- and omics-based platforms discussed above provide unprecedented molecular resolution, they share an inherent dependence on labeling, fixation, or optical reporters that can perturb NM physicochemical properties. This is a particular concern for inorganic NMs, which often lack intrinsic fluorescence and whose surface chemistry, protein corona, and agglomeration state can be altered by fluorophore conjugation. Compounding this, conventional viability assays, including XTT, MTT, LDH, and ATP-based formats, can be affected by NM optical absorbance, light scattering, catalytic activity, or adsorption of assay reagents, leading to false-positive or false-negative cytotoxicity readouts [38, 130].

Label-free enhanced dark-field imaging (EDF) addresses these gaps by detecting NMs through their intrinsic light-scattering signatures rather than added reporters. Under oblique illumination, only light scattered by the sample is collected, and for plasmonic NMs, this scattering is amplified by localized surface plasmon resonance (LSPR), enabling single-particle detection of metallic NPs as small as approximately 10 nm. When EDF is coupled with hyperspectral imaging, the resulting enhanced dark-field hyperspectral imaging (EDF-HSI) platform yields a full visible-to-near-infrared scattering spectrum for each pixel, allowing spectral angle mapping or spectral feature fitting to discriminate NM identity, size distribution, agglomeration state, and corona-induced spectral shifts in situ [131]. Because the technique is non-destructive and requires no staining, it is compatible with live cells, ex vivo tissue sections, and 3D culture systems.

Several studies illustrate the value of EDF-HSI within NAM workflows. Vetten et al. used EDF-HSI to confirm cellular uptake of citrate-stabilized AuNPs across three cell lines while bypassing AuNP-induced interference with XTT- and ATP-based viability assays, decoupling uptake from toxicity [130]. Shannahan et al. resolved corona-dependent shifts in AgNP scattering spectra and linked them to altered macrophage uptake, demonstrating how HSI can capture changes in the biological identity of NMs that may be difficult to resolve using fluorescence-based methods [132]. The label-free uptake–toxicity decoupling enabled by EDF-HSI has been reproduced independently across multiple cell types and particle chemistries, including AuNP uptake and localization cross-validated against X-ray nanotomography in plant roots [133], nanoplastic accumulation visualized and quantified in living human cells by enhanced dark-field hyperspectral imaging [134], and carbon nanodot nuclear penetration tracked in A549 cells [135]. Mortimer et al. extended the approach to non-plasmonic NMs, semi-quantitatively mapping TiO₂, CuO, and quantum-dot uptake in protozoa, of relevance to eco-NAMs [136]. Mercer et al. applied EDF to track inhaled multi-walled carbon nanotubes (MWCNTs), CeO₂, and diesel exhaust particles in lung tissue sections, providing in vivo–ex vivo connectivity for inhalation hazard assessment [137]. Beyond particle localization, EDF-HSI with plasmonic nanoprobes has also been applied to quantitative single-cell molecular mapping [138], and the approach has been extended to non-plasmonic and biological nanostructures such as self-assembling viral nanoparticles [139], underscoring the breadth of its label-free applicability. Building on these advances, Baek et al. employed reflected dark-field microscopy with time-lapse and Z-stack acquisition to follow AuNP internalization in live mouse liver organoids. This study showed that the ECM dome of conventional organoid culture can physically restrict NP access, a systematic bias addressed by their high-throughput-compatible floating-organoid platform [68].

Remaining challenges include limited spectral specificity for weakly scattering polymeric NMs, discrimination from endogenous scatterers (vesicles, lipid droplets), and the need for harmonized reference materials and analysis pipelines to support regulatory acceptance. Correlative implementations with transmission electron microscopy and Raman spectroscopy, together with metrology-driven standardization, are progressively addressing these gaps and consolidating EDF-HSI as a complementary, NM-specific readout within high-tier NAM platforms.

### In silico NAMs

As high-throughput screening (HTS) and high-content imaging generate large, multidimensional toxicological datasets, in silico methods have become increasingly important for interpreting hazard signals and prioritizing NMs for further testing [37, 140, 141]. The field of in silico nanotoxicology has expanded beyond descriptor-based QSAR models to include AI-driven platforms that integrate physicochemical descriptors and biological data to predict toxicity-related outcomes. In parallel, mechanistic modeling approaches such as PBK models provide a complementary framework for linking external exposure, internal dose, and toxicokinetic behavior to risk assessment. Accordingly, this section considers in silico NAMs as a three-layered framework comprising data-driven prediction, kinetic translation, and mechanistic pathway integration. Representative in silico NAM platforms relevant to nanotoxicology, together with their typical formats, key outputs, strengths, and limitations, are summarized in Table 4.

**Table 4:** Overview of in silico NAM platforms for nanotoxicology

NAM platform	Typical format	Main use in nanotoxicology	Key outputs	Main strengths	Main limitations
Nano-QSAR/QNAR models	Descriptor-based models using NM physicochemical properties, structural descriptors, and experimental endpoints	First-tier toxicity prediction, material grouping, read-across support, hazard prioritization	Predicted cytotoxicity, genotoxicity, inflammatory potential, hazard class, descriptor–endpoint relationships	Interpretable, relatively low-cost, useful for screening and prioritization	Requires high-quality harmonized descriptors; limited by applicability domain and data scarcity
Classical machine learning models	Random forests, support vector machines, gradient boosting, and related supervised learning models	Prediction of NM toxicity endpoints from curated physicochemical and biological datasets	Toxicity classification, endpoint prediction, feature importance, uncertainty estimates	Handles nonlinear relationships; compatible with structured nanosafety datasets	Performance depends strongly on dataset quality, external validation, and endpoint consistency
Deep learning and multitask models	Neural networks, graph-inspired models, multitask learning, and high-dimensional data integration models	Prediction across multiple related endpoints, data-gap filling, integration of heterogeneous data streams	Multi-endpoint toxicity predictions, latent patterns, cross-endpoint inference	Can model complex high-dimensional relationships and related endpoints	Less interpretable; requires larger datasets; prone to overfitting in sparse nanosafety datasets
Generative AI and inverse-design models	Generative models, GANs, variational models, and design–prediction workflows	Design or prioritization of safer NMs with desired functionality and reduced hazard potential	Candidate material structures, predicted safety profiles, optimized descriptor spaces	Supports proactive safer-by-design material development	Requires careful constraint setting, experimental confirmation, and transparent validation
AutoML platforms	Automated workflows for preprocessing, algorithm selection, hyperparameter tuning, and model comparison	Democratized nanotoxicity model development and benchmarking	Optimized predictive models, performance metrics, selected descriptors, ranked algorithms	Improves accessibility for non-specialists; supports systematic model comparison	May obscure modeling decisions; still requires expert curation, applicability-domain assessment, and external validation
NLP/LLM-assisted data extraction	Literature-mining pipelines, text-mining workflows, LLM-assisted extraction and curation	Rapid collection of nanotoxicity data from unstructured literature to expand training datasets	Extracted NM descriptors, exposure conditions, endpoints, assay metadata, curated datasets	Accelerates dataset generation; helps address fragmented literature data	Risk of extraction errors, incomplete metadata, unsupported associations, and inconsistent reporting standards
Nanoinformatics data infrastructures	eNanoMapper, NanoDatabank, OCHEM, Nanoparticle Information Library, FAIR-aligned databases	Storage, harmonization, sharing, and reuse of NM physicochemical and toxicological data	Curated datasets, ontologies, metadata, model-ready descriptors	Enables reproducibility, interoperability, and model development	Data incompleteness, inconsistent metadata, and variable experimental quality remain major bottlenecks
PBK/QIVIVE models	PBK models linked to IVIVE/QIVIVE workflows	Translation of in vitro bioactivity and delivered dose to internal exposure and tissue-relevant dose metrics	Tissue concentrations, biodistribution, clearance, internal dose, bioactivity–exposure relationships	Provides quantitative bridge between in vitro NAMs and human-relevant exposure assessment	NM-specific processes such as agglomeration, dissolution, corona formation, phagocytosis, and slow clearance are difficult to parameterize
AOP-informed toxicogenomic and network models	Omics integration, network toxicology, AOP mapping, key-event enrichment, pathway-based dose–response modeling	Mechanistic interpretation of NM-induced perturbations and linkage to regulatory-relevant outcomes	MIE/KE/AO mapping, pathway perturbation, mechanism-of-action signatures, key-event dose metrics	Connects high-dimensional NAM data to mechanistic hazard interpretation and NGRA	Requires biologically relevant test systems, robust omics pipelines, and careful separation of adaptive versus adverse responses
Integrated digital twin models	Hybrid experimental–computational models combining NAM data, PBK, AI, and systems biology	Simulation of exposure scenarios, dynamic tissue responses, and SSbD-oriented safety assessment	Time-resolved response trajectories, virtual exposure outcomes, scenario-specific risk predictions	Enables iterative prediction–testing cycles and systems-level integration	Emerging concept; requires extensive validation, standardized inputs, and regulatory confidence

#### Data-driven approaches

Within this framework, data-driven approaches play a central role in nanosafety evaluation by identifying predictive relationships within physicochemical and biological datasets. These approaches span descriptor-based nano-quantitative structure–activity relationship (nano-QSAR) and quantitative nanostructure–activity relationship (QNAR) models, machine learning (ML), generative AI, and automated literature-mining workflows, supported by standardized nanoinformatics resources and regulatory expectations for validation and interpretability.

At the descriptor-based end of this spectrum, the adaptation of classical QSAR principles to NMs, giving rise to nano-QSAR and QNAR modeling, represents a significant conceptual and technical evolution [142]. Unlike conventional small molecules, whose structures can often be represented by molecular graphs or by Simplified Molecular Input Line Entry System (SMILES), a one-dimensional string notation for encoding chemical structures, NMs require nano-specific descriptors that capture particle-level properties such as size, surface area, surface charge, shape, crystallinity, agglomeration state, and surface chemistry [35, 123, 124, 142]. Nano-QSAR models have been developed for endpoints including cytotoxicity, genotoxicity, and inflammatory potential in metal oxide NMs, with band gap energy, cation charge, and specific surface area emerging as recurrent predictive descriptors across model families [124–127, 143]. These modeling efforts are supported by eNanoMapper, an open-access, ontology-driven infrastructure for storage, sharing, and computational modeling of NM physicochemical and toxicological data that has been integrated with automated ML workflows for nanosafety evaluation [36, 144]. NanoDatabank, the Online Chemical Modeling Environment (OCHEM), and the Nanoparticle Information Library further contribute to the nanoinformatics ecosystem [145, 146].

Building on these curated descriptor spaces, ML algorithms, including random forests and support vector machines, dominate the current market for predictive toxicology because of their interpretability and ability to handle structured descriptor–endpoint datasets. More recently, the field has witnessed the emergence of generative AI and deep learning architectures. Generative AI, such as generative adversarial networks (GANs), enables the proactive design of novel materials or compounds with predefined safety profiles, shifting toxicology from a reactive screening discipline to a proactive design science. These advanced models can analyze complex, high-dimensional data and support prediction across multiple related endpoints, although recent comparative work shows that multitask model performance may vary substantially across algorithms and datasets, underscoring the importance of systematic benchmarking and fit-for-purpose model selection [147].

Predictive modeling for nanosafety is notoriously challenged by the extreme heterogeneity, data sparsity, and high-dimensional nature of nanomaterial datasets, where subtle variations within a single nanoform class can lead to drastically disparate toxicological outcomes [35, 36]. To democratize the use of AI in nanosafety, Automated Machine Learning (AutoML) platforms (e.g., Vertex AI, Azure, Dataiku) have been developed to automate the model development pipeline, from data preprocessing to algorithm selection and hyperparameter tuning [140, 148]. Comparative studies indicate that AutoML-generated models often outperform conventional, manually tuned ML models in nanotoxicity prediction by providing higher accuracy, precision, and recall [140].

In nanotechnology, AutoML is valuable because it enables systematic comparison of models across high-dimensional, heterogeneous descriptor spaces linking material properties, exposure conditions, and biological endpoints. These spaces are especially relevant to nanoforms, whose descriptors include size distribution, surface charge, crystal phase, coating-dependent agglomeration, dissolution, and other context-dependent particle-level features. AutoML lowers the technical barrier to model development and hazard prioritization, but does not replace expert data curation, descriptor harmonization, endpoint consistency, applicability-domain assessment, or external validation. This balance is essential for accessible, scientifically transparent predictive tools [36, 149, 150].

Because nanosafety data remain fragmented across heterogeneous and often unstructured literature sources, large language models (LLMs) and natural language processing (NLP) frameworks such as LangChain are therefore being deployed to extract nanotoxicity data from unstructured literature and rapidly curate high-quality datasets, significantly accelerating training-set development [141]. For regulatory applications, however, data-driven models require descriptor harmonization, data quality, applicability-domain definition, external validation, uncertainty characterization, transparent documentation, and, where possible, explainable outputs.

#### Applications of PBK models to nanomaterials

Whereas data-driven models primarily infer descriptor–endpoint relationships, PBK models provide the kinetic-translation layer needed to relate external exposure to internal dose in regulatory toxicology [151–153]. Originally developed for pharmaceutical applications, PBK models have since been applied in chemical risk assessment, predominantly for small molecules, although related approaches have also been explored for larger molecules [154–157].

Extending PBK models to NMs, however, poses a distinct and complex challenge because NMs encompass a broad spectrum of materials, ranging from soluble or degradable forms to poorly soluble or persistent solid particles [158–160]. NM biological behavior is influenced, among other factors, by surface coating, primary particle size, chemical composition, and aggregation kinetics, all of which can affect protein corona formation, bioavailability, and biodistribution. Modeling the disposition of NMs in vivo therefore requires unique adaptations that go beyond classical PBK frameworks. Key NM-specific processes that should be incorporated into PBK modeling include phagocytic uptake, which strongly influences biodistribution, particularly in the lung, spleen, and liver, and slow and non-linear clearance kinetics, which can lead to long-term tissue sequestration [158–160]. In addition, dynamic agglomeration can alter particle size, surface area, morphology, and effective density under organ-specific conditions such as pH [159].

These modeling challenges are compounded by fundamental limitations in toxicokinetic studies. As reported by Chen et al. plasma toxicokinetics are often measured at only a few time points, precluding robust estimation of maximum plasma concentration [37]. In vitro systems can show differences of up to six orders of magnitude between the administered dose and the delivered cellular dose because of NM agglomeration, sedimentation, and diffusion. If unaddressed, this discrepancy may propagate directly into model predictions and undermine IVIVE. Together, these considerations make PBK modeling particularly important for regulatory NAM workflows, where it provides a quantitative bridge between in vitro bioactivity, delivered dose, and human-relevant exposure metrics.

#### From toxicogenomics to AOPs: integrating multi-omics for mechanistic hazard interpretation

Traditional toxicological assessment is chemocentric, linking adverse effects primarily to chemical properties, structure, and dose, often neglecting the biological system in which the exposure occurs. This view is increasingly complemented by an alternative perspective, which emphasizes that exposure outcomes are also shaped by the molecular state of the biological system, represented by the expressed genes and epigenetic modifications present in a certain homeostatic stage [161]. In this context, toxicogenomics (TGx) has emerged as a well-established framework that uses omics data to characterize the effects of chemicals and materials [162–164]. Recent advances in TGx suggest that toxicity can emerge from multiscale interactions between a chemical or material and the exposed biological system. These interactions can be organized into a structured, causal and multiscale process that describes the mechanism of action (MOA) of a compound. Within the Adverse Outcome Pathway (AOP) framework, MOAs are represented as causal chains linking molecular initiating events (MIEs) to adverse outcomes (AOs) [165, 166]. This paradigm shifts TGx beyond the interpretation of gene lists, enabling the alignment of molecular perturbations with higher-level biological endpoints [167]. In this context, the molecular state of the exposed system, including gene expression, regulatory context, and epigenetic features, determines how chemical- or nanomaterial-induced perturbations propagate across biological scales. This concept helps explain variability of a chemical response across different systems and the importance of choosing the relevant in vitro model to characterize hazards [168]. Notably, del Giudice et al. revealed a conserved molecular response to nanomaterial particulates across species, suggesting that evolutionarily conserved mechanisms coexist with system-specific variability [169]. This cross-species conservation supports the application of TGx within a One Health framework, allowing for the integration of evidence across humans, animals, and the environment [165, 166].

While TGx experiments using in vitro NAMs provide valuable insights into the MOAs of chemicals and materials, individual models capture only context-dependent fragments of the overall mechanisms. Moreover, different in vitro systems may activate distinct portions of the same MOA, meaning that each model reflects only a subset of the full mechanistic landscape. Consequently, integrative approaches, based on the AOP framework and network-informed models, are required to reconstruct these partial views and combine them with in silico approaches such as PBK models or QSAR [170–172]. This underscores the need to define the applicability domain (AD) of a test system in terms of which mechanistic events and pathways it can express in response to an exposure. This is reflected in the presence of molecular machinery that enables chemical interaction through appropriate receptors and subsequently propagates it through the relevant multiscale mechanisms. To estimate this AD, the molecular makeup of the test system must be meticulously characterized across different compartments or scales; multi-omics approaches can inform the potential ability of a specific biological system to respond to a chemical and exert a specific mechanism [161].

Regarding nanomaterials, this characterization must extend to the dynamic behavior of the material itself, considering the nano–bio interface. Namely, the protein corona around the nanomaterial redefines the interactions that it can have with biological receptors in the cell [173, 174] and therefore heavily determines which MIEs are implicated in a given test system. While it remains to be established whether TGx-to-AOP approaches have the resolution necessary to distinguish between biologically distinct corona compositions, mechanistic TGx approaches have previously shown the ability to distinguish distinct transcriptomic signature caused by differing nanomaterial physical and surface properties, including fiber geometry, surface area, aspect ratio and colloidal state, both at the level of individual exposures [175–177] and when leveraged to derive grouping across larger nanomaterial libraries [178]. Another challenge remains that in vitro media-based nanomaterial preparations can result in corona compositions that differ substantially from those formed in vivo in plasma. A possible strategy to mitigate this limitation is mass spectrometry-based quantification of the in vitro intracellular uptake of the nanomaterial and comparison to in vivo cellular uptake, whereby comparable intracellular concentrations can lead to human-relevant mechanistic modeling. This can be particularly important in cases such as frustrated phagocytosis, where substantial extracellular or cell-associated material may be present despite limited internalization; measuring cell-associated and, where relevant, intracellular or cytosolic fractions can therefore provide more realistic dose metrics for mechanistically grounded TGx interpretation through the AOP framework [179]. This concept is also applicable to agglomeration of NM, a phenomenon that can significantly alter the delivered cellular dose with respect to the nominal administered concentration [180].

Moreover, transformation processes such as dissolution and degradation determine whether the resulting transcriptomics signature reflects a particulate exposure or ion-mediated mechanisms [174]. Previous studies highlight differences between highly soluble NMs (e.g. ZnO) versus poorly soluble ones (e.g. NiO and CuO), highlighting the need to characterize dissolution prior to mechanistic interpretation of transcriptomic data [181–183].

It is important to interpret molecular perturbations within the boundaries and context of the test system, rather than treating them as absolute indicators of hazard, because the toxicogenomic signature of a system describes how it adapts to chemical exposure, not the full toxicological mechanism. Therefore, it is necessary to distinguish between system-specific molecular responses and toxicologically relevant mechanistic signals [184].

Moreover, network toxicology provides a way to translate and integrate high-dimensional omics readouts into a system-level mechanistic representation that recapitulates the interactions between chemicals and exposed systems. Rather than interpreting toxicogenomics data as a list of altered genes, this approach infers networks that describe molecular perturbations, facilitating links to key events (KEs) and ultimately the description of a system-level MOA [168, 172, 185]. When combined with dose–response modeling and AOP-informed key event enrichment [170, 177, 186], this approach enables not only the identification of mechanistic pathways but also their quantitative characterization in terms of sensitivity and temporal dynamics, while highlighting that mechanistic patterns and the grouping of exposures emerge from the combined influence of chemical composition, system features, and exposure conditions rather than any single factor alone [178]. Tying TGx to the AOP framework, while a recently developed strategy, is being used successfully across the field of chemical safety assessment, both in targeted approaches, for example, for nanomaterials and endocrine disruptors [187, 188], but also in whole AOP network approaches allowing any compound of interest to be broadly analysed across the network [168, 189]. Such an AOP-anchored omics approach will only improve for nanomaterial safety assessment as successive iterations of the AOP network evolve, and the corresponding gene annotations are refined.

Altogether, these concepts support a threefold strategy that integrates chemical properties, biological system characteristics, and the AOP framework. Chemical properties identify the space of MIEs and mechanistic events, biological system characteristics delimit which of these events can be realized, and AOPs provide the structured causal chains needed to map observed molecular responses to regulatory-relevant outcomes. This integrated framework enables a mechanistically grounded and biologically contextualized interpretation of hazard, moving beyond the isolated use of NAM-derived evidence [161].

## Global regulatory landscape: harmonization and divergence

The global regulatory landscape for toxicological safety assessment is undergoing a paradigm shift, moving from historical reliance on vertebrate animal models toward fit-for-purpose, human- or target-species-relevant NAMs that are reproducible and suitable for defined regulatory contexts. These approaches span advanced in vitro systems, in chemico assays, omics-based readouts, in silico models, PBK modeling, and integrated evidence frameworks. For nanotechnology, this shift is especially consequential because nanomaterial hazards cannot be inferred from chemical composition alone. Material properties, transformation processes, exposure conditions, and delivered dose also shape biological responses and regulatory interpretation [35, 37, 123, 190, 191].

This creates a dual regulatory challenge. First, NAMs must be demonstrated to be biologically relevant, reproducible, technically robust, and fit for purpose within a clearly defined context of use [10, 67, 89, 192]. Second, nanomaterial testing requires parallel standardization of physicochemical characterization, sample preparation, dispersion protocols, exposure conditions, and dosimetry [35, 37, 190, 191]. Consequently, regulatory acceptance of NAMs for nanomaterial assessment depends not only on biological method validation but also on reliable characterization of the test material and exposure scenario. Accordingly, the regulatory discussion below is organized around the points at which NAM-derived evidence becomes decision-relevant for nanomaterials: nanoform identity, dispersion and sample-preparation protocols, exposure scenario, delivered-dose metrics, data integrity, and integration of biological evidence with physicochemical characterization data.

**Fig. 3:**
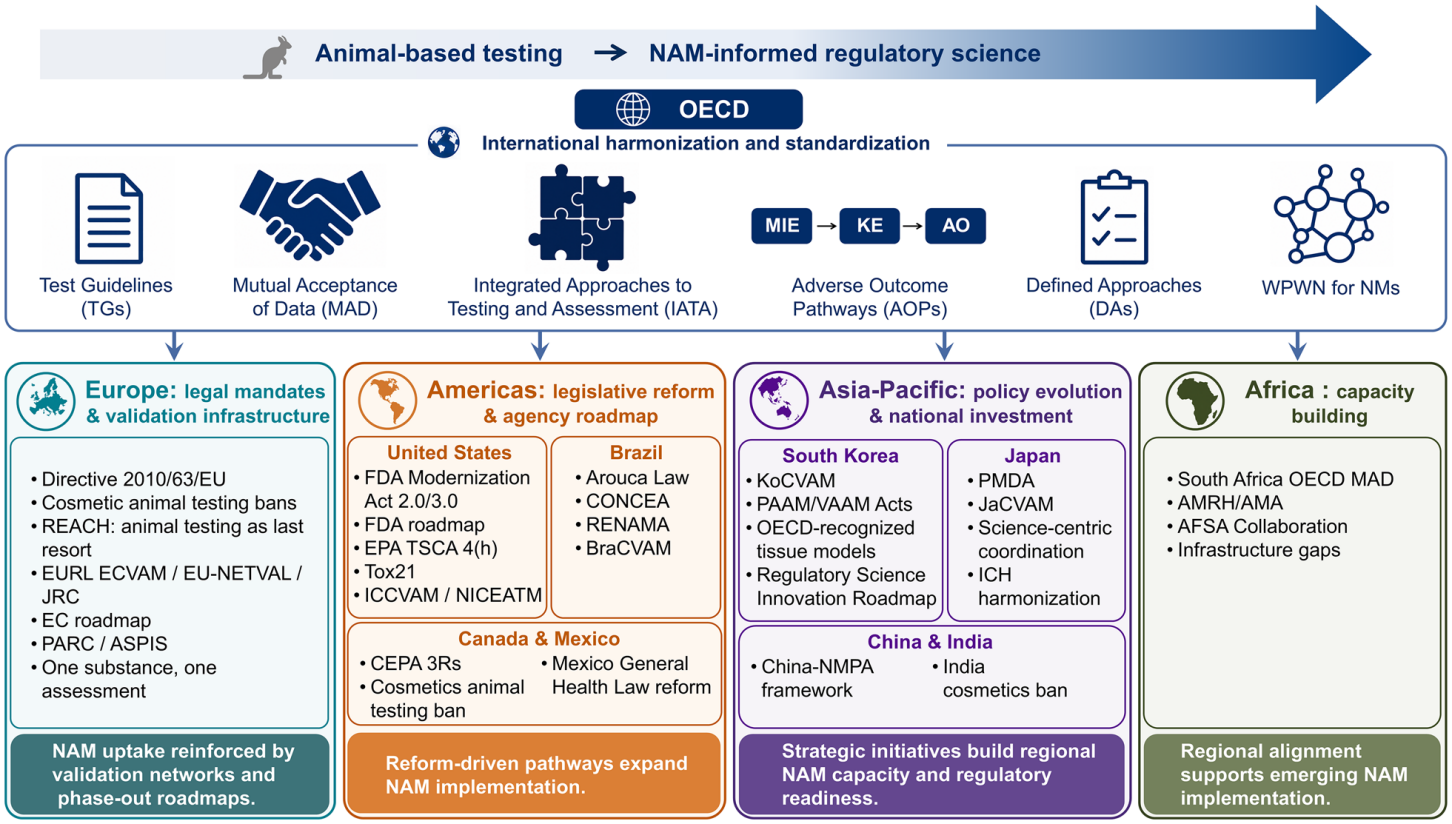
Global regulatory landscape and implementation pathways for NAM-informed regulatory science

At the global level, harmonization is supported primarily by the OECD Test Guidelines Programme, the Mutual Acceptance of Data (MAD) system, and OECD Guidance Documents on nanomaterial testing [67, 190, 193, 194]. Regional frameworks in the European Union (EU), Africa, North America, South America, the Asia–Pacific region, and other jurisdictions differ in the pace and extent of NAM implementation. However, they increasingly align around common principles: reducing unnecessary animal testing, validating methods for defined contexts of use, transparent reporting, and integrating multiple lines of evidence [9, 16, 19, 195–229]. Accordingly, this section focuses primarily on human health regulation while noting ecotoxicological NAMs (Eco-NAMs) where environmental fate and bioavailability affect nanomaterial hazard assessment [40–42, 117, 191, 230–234]. The global transition from animal-based testing toward NAM-informed regulatory science, including international harmonization frameworks, regional implementation pathways, and shared determinants of NAM uptake, is summarized in Fig. 3.

### The OECD Framework

Because fragmented regulatory requirements can impede the global adoption of NAMs, international standard-setting bodies—most notably the OECD—play a central role in harmonization [9, 10].

The OECD advances NAM integration through its Test Guidelines Programme, while the MAD system provides a framework for accepting data generated in accordance with OECD Test Guidelines (TGs) and Good Laboratory Practice (GLP) [193]. Under MAD, data generated in one participating jurisdiction in accordance with OECD TGs and GLP must be accepted by other participating jurisdictions, thereby promoting regulatory harmonization and reducing redundant testing [193]. In recent years, the OECD has expanded its portfolio of non-animal and alternative-method TGs for selected endpoints, including reconstructed human epidermis models for skin corrosion and irritation, such as TG 431 and TG 439 [235, 236], as well as in vitro and in chemico methods for skin sensitisation, including Direct Peptide Reactivity Assay (DPRA)-type assays under TG 442C, KeratinoSens™-type assays under TG 442D, and human Cell Line Activation Test (h-CLAT)-type assays under TG 442E [23, 237–239]. These assays can also be combined within AOP-informed defined approaches, such as TG 497 for skin sensitisation [23]. Nevertheless, these examples represent endpoint-specific successes rather than a comprehensive replacement of animal-based testing. Moreover, the OECD has published initial recommendations for assessing developmental neurotoxicity (DNT) using 17 NAMs compiled within the DNT in vitro battery (DNT IVB) [240–242]. The DNT IVB is composed mainly of human cell-based NAMs that model eight key processes of brain development and have been characterized in terms of their biological and chemical applicability domains [242–244]. A substantial proportion of OECD TGs still rely on animal-based or whole-organism studies, and NAM uptake remains uneven, particularly for complex systemic endpoints requiring repeated-dose, reproductive, developmental, toxicokinetic, or multi-organ data [9, 10].

For nanomaterials, OECD harmonization is particularly important because regulatory interpretation depends not only on biological hazard endpoints but also on particle characteristics, surface properties, dispersion stability, delivered dose, dissolution, agglomeration, and exposure route [35, 37, 190, 191]. Accordingly, several OECD TGs and supporting guidance documents are directly relevant to nanomaterial characterization, exposure assessment, inhalation toxicity, ecotoxicity, and NAM-based hazard evaluation, as summarized in Table 5.

**Table 5:** Selected OECD TGs and guidance documents relevant to nanomaterial assessment

OECD TG	Endpoint/purpose	Relevance for nanomaterials	Key consideration
TG 124	Volume-specific surface area	Nano-specific descriptor for material identity, grouping, read-across, and hazard interpretation	Interpret with size, density, porosity, and surface chemistry
TG 125	Particle size and size distribution	Core nano-specific characterization TG	Sample preparation and method selection depend on shape, agglomeration, and dispersibility
TG 126	Hydrophobicity index	Surface-property descriptor relevant to dispersion, corona formation, uptake, and fate	Classical log Kₒw is not applicable to particulate materials
TG 318	Dispersion stability	Supports exposure interpretation and test-system design	Strongly affected by pH, ionic strength, proteins, organic matter, and agglomeration
TG 403	Acute inhalation toxicity	Relevant for acute inhalation hazard, but not nano-specific	May miss persistence, delayed inflammation, and lung-burden effects
TG 412	28-day inhalation toxicity	Relevant for repeated inhalation exposure to NMs	Requires aerosol characterization, deposited dose/lung burden, and pulmonary endpoints
TG 413	90-day inhalation toxicity	Relevant for subchronic effects of persistent, poorly soluble, or fibrous NMs	Requires exposure characterization, bronchoalveolar lavage/histopathology, clearance, and lung burden
TG 417	Toxicokinetics	Relevant for biodistribution and internal exposure interpretation	Particle uptake, tissue sequestration, dissolution, and clearance kinetics must be considered
TG 431/439	Skin corrosion and irritation using reconstructed human epidermis	Relevant for nano-enabled topical products	Check penetration, formulation effects, and assay interference
TG 442C/D/E; TG 497	Skin sensitisation assays and defined approaches	Case-by-case relevance for nanoforms	Assay interference and reduced bioavailability may limit applicability
TG 487	In vitro micronucleus test	Relevant for chromosomal damage assessment	Requires uptake verification, cytotoxicity control, and interference assessment
TG 489	In vivo comet assay	Relevant where in vitro genotoxicity evidence is insufficient	Tissue exposure and biodistribution are critical
TG 249	RTgill-W1 fish cell line acute toxicity	Eco-NAM included for cross-domain relevance	Useful example of validated cell-line-based alternative testing

These nano-relevant TGs illustrate that regulatory acceptance of NAMs for nanomaterials requires parallel standardization of both the biological test system and the material/exposure characterization. Even technically robust NAM data may remain difficult to compare across laboratories or interpret consistently across jurisdictions if material preparation, exposure characterization, dose metrics, transformation reporting, and data-quality controls are not harmonized. OECD guidance documents therefore play an important complementary role by translating these standardization needs into practical recommendations for dispersion preparation, dosimetry, inhalation exposure design, aerosol or medium characterization, and interpretation of nanomaterial-specific behavior [35, 37, 190, 191, 194, 245].

To support regulatory decision-making through the integration of diverse evidence sources, particularly where single NAMs are insufficient to capture complex in vivo organismal responses, the OECD champions IATA [12, 20, 21, 246]. IATA uses structured approaches to integrate, evaluate, and weigh diverse evidence streams—including existing human data, in vitro assays, and in silico predictions—often guided by AOPs [12, 246]. By anchoring NAMs to AOPs, regulators can evaluate safety using a mechanistic understanding of toxicity rather than relying solely on apical endpoints. Furthermore, the OECD has developed Defined Approaches (DAs), which formalize rule-based procedures for combining multiple NAMs to support regulatory classifications while improving consistency, transparency, and reproducibility [15, 22, 23].

Beyond OECD-led harmonization, sector-specific guidance from the International Council for Harmonisation (ICH) supports pharmaceutical safety assessment, including the qualification and potential use of alternative assays under ICH S5(R3) and model-informed drug development under ICH M15. These efforts are especially relevant to PBK modeling, in silico NAMs, and the use of NAM-derived evidence in pharmaceutical regulatory submissions.

More recently, the OECD Working Party on Hazard Assessment has been developing a NAM-based NGRA framework, building on the use of NAM-based bioactivity–exposure ratios and related international efforts, including the Alternative Safety Profiling Algorithm (ASPA) framework [247].

In parallel, the OECD advances nanomaterial safety assessment through the Working Party on Manufactured Nanomaterials (WPMN) [190]. These efforts include adapting existing TGs and supporting nano-specific in vitro and in silico approaches for advanced-material safety assessment, consistent with SSbD principles [190, 248]. OECD harmonization is also relevant to ecotoxicological NAMs, where dispersion stability, dissolution, sedimentation, and bioavailability affect environmental exposure and the interpretation of cell-based Eco-NAMs [40–42, 117, 191].

### Europe

The EU has historically been one of the leading jurisdictions in the integration of non-animal testing alternatives, using stringent legislation to actively drive innovation in regulatory risk assessment. This commitment is rooted in Article 13 of the Treaty on the Functioning of the EU, which recognizes animals as sentient beings [201]. Directive 2010/63/EU further operationalizes the 3Rs by requiring scientifically satisfactory non-animal methods or testing strategies to be used wherever possible [17]. Within this framework, NAMs are not merely optional tools but a regulatory expectation, particularly in sectors where robust alternatives have emerged [16, 206, 207, 209, 249, 250].

An important driver of NAM adoption was the EU Cosmetics Directive, which instituted sequential bans on animal testing: first on animal testing for finished cosmetic products (2004), then for cosmetic ingredients (2009), culminating in a complete marketing ban on animal-tested cosmetics in 2013 [250]. These measures effectively tied market access to the use of non-animal methods, compelling the cosmetics sector to become an early adopter and major investor in advanced in vitro systems, including reconstructed human tissue models (such as EpiSkin™) and non-animal screening batteries [206, 207, 251]. This experience also laid important scientific and regulatory foundations for broader application of NAMs, including in cosmetic ingredients containing NMs.

In parallel, the chemicals legislation REACH (Registration, Evaluation, Authorisation and Restriction of Chemicals) explicitly positions vertebrate animal testing as a last resort [209]. Registrants must justify any proposed animal study and systematically consider existing information and alternative approaches, including data sharing, read-across, QSAR and other in silico models, and in vitro data, before resorting to new animal tests. This legal architecture has incentivized the development and use of NAMs, including approaches adapted to the specific characteristics of NMs, such as nano-QSAR and nano-specific in vitro assays [209]. For nanoform registration and evaluation under frameworks such as REACH, the critical issue is not only whether a non-animal method is available, but whether the NAM-derived evidence can be linked to a well-defined nanoform, including particle size distribution, surface chemistry, coating, dissolution or agglomeration behavior, and exposure-relevant dose metrics. However, practical implementation remains endpoint-dependent, with NAM uptake being more established for selected local or short-term endpoints than for complex systemic endpoints. This has contributed to broader EU efforts, including the “one substance, one assessment” approach, to improve coherence, data sharing, and the avoidance of unnecessary duplicate testing across chemical regulations.

To ensure scientific robustness and regulatory credibility, the European Centre for the Validation of Alternative Methods (ECVAM) was established within the European Commission’s Joint Research Centre (JRC) in 1991 and later became the EU Reference Laboratory for Alternatives to Animal Testing (EURL ECVAM). Together with the European Union Network of Laboratories for the Validation of Alternative Methods (EU-NETVAL), EURL ECVAM supports method validation, standardization, and regulatory uptake of alternative approaches, including their integration into OECD test guidelines [23, 210, 237, 249, 252]. This infrastructure is increasingly relevant for complex, mechanistically anchored NAMs in nanotechnology, such as advanced in vitro models and integrated testing strategies [253].

The EU regulatory trajectory is also strongly shaped by public expectations. Following the European Citizens’ Initiative “Save cruelty-free cosmetics—Commit to a Europe without animal testing” [211], the European Commission issued Communication C(2023)5041 and committed to developing a comprehensive “Roadmap Towards Phasing Out Animal Testing for Chemical Safety Assessment” by early 2026 [16]. The roadmap is being shaped by dedicated working groups on Human Health, Environmental Safety Assessment, and Change Management, tasked with defining concrete milestones and transition mechanisms in collaboration with regulators, industry, and civil society [16]. These efforts are supported by EU-funded and partnership-based initiatives, such as PARC [212], involving numerous Member States, and the ASPIS cluster [213]. Beyond EU-level programmes, initiatives such as the Netherlands-based Ombion Centre for Animal-Free Biomedical Translation and Roche’s Institute of Human Biology in Basel illustrate how national and industry-led efforts are also advancing human-relevant model systems, validation support, and translational implementation [254, 255]. Within this ecosystem, NAMs in nanotechnology—spanning advanced in vitro models, high-content analytics, and in silico approaches—are increasingly positioned as important tools for delivering mechanistically informed safety evaluations aligned with European policy and innovation goals.

### North America

Historically, regulatory frameworks across North America have relied heavily on mandatory animal data to evaluate chemical and pharmaceutical safety. Today, the region is undergoing a transition from historical animal mandates toward the broader adoption of NAMs. While the United States has enacted landmark legislative reforms and established dedicated validation centers, broader regional harmonization efforts are gradually drawing in nations like Canada and Mexico.

#### The United States: legislative overhauls and agency roadmaps

The United States has recently expanded its role in NAM implementation through legislative reforms and agency roadmaps [256–258].

A major regulatory change occurred with the passage of the FDA Modernization Act 2.0 in December 2022, which explicitly removed the 80-year-old federal mandate that required animal testing for new drug development [256]. FDA Modernization Act 3.0 has been proposed to align legacy FDA regulations with the broader “nonclinical” framework introduced by FDA Modernization Act 2.0 [259]. To operationalize these powers, the FDA rolled out a comprehensive "Roadmap to Reducing Animal Testing in Preclinical Safety Studies," initially focusing on monoclonal antibodies (mAbs) [257]. Because animals often fail to predict human-specific cytokine storms, the FDA is supporting the use of human in vitro assays and organ-on-chip platforms [257, 260–262]. Through the Innovative Science and Technology Approaches for New Drugs (ISTAND) pilot program, the FDA accepted the first letter of intent for a human Liver-Chip drug development tool designed to assess drug-induced liver injury (DILI) risk, marking an early step in the drug development tool qualification pathway rather than full qualification [262, 263]. Furthermore, in March 2026, the FDA released a draft guidance establishing four core validation principles for NAMs: 1) Context of Use, 2) Human Biological Relevance, 3) Technical Characterization, and 4) Fit-for-Purpose [258].

In parallel, the U.S. Environmental Protection Agency (EPA) is advancing NAM adoption under the amended Toxic Substances Control Act (TSCA) Section 4(h), which explicitly requires the agency to reduce and replace vertebrate animal testing. The EPA has set clear targets to reduce mammalian study requests by 30% by 2025 and eliminate them entirely by 2035 [195, 264]. In this context, Tox21 is a broader federal collaboration involving the National Institute of Environmental Health Sciences (NIEHS), the National Center for Advancing Translational Sciences (NCATS), the FDA, and the EPA, rather than an EPA-only initiative [5]. U.S. National Institutes of Health (NIH) activities also support NAM development through programs such as the NCATS Tissue Chip for Drug Screening program and the NIH Common Fund Complement Animal Research In Experimentation (Complement-ARIE) program, which respectively advance human cell-based MPS and the development, standardization, validation, and use of human-based NAMs [265]. These efforts are further coordinated through Interagency Coordinating Committee on the Validation of Alternative Methods (ICCVAM), with support from the NTP Interagency Center for the Evaluation of Alternative Toxicological Methods (NICEATM), to facilitate cross-agency evaluation and implementation of alternative test methods [266].

#### Canada and Mexico

Although the United States has been a major influence on regulatory developments in North America, the adoption of NAMs across the region is being shaped by both national reforms and international cooperation [267–269]. In Canada, Health Canada and Environment and Climate Change Canada have explicitly incorporated NAMs into chemical risk assessment under the Canadian Environmental Protection Act (CEPA), and recent CEPA-related reforms have emphasized the replacement, reduction, and refinement of vertebrate animal testing; Canada also enacted a ban on cosmetic animal testing in 2023 [267, 268]. A prominent example of international coordination is the International Cooperation on Cosmetics Regulation (ICCR), a voluntary group of cosmetics regulatory authorities from Canada, the United States, Brazil, the European Union, Japan, and several other jurisdictions that meets to discuss common issues in cosmetics safety and regulation while engaging in dialogue with relevant industry trade associations [269]. Mexico has also adopted a regulatory reform through its 2021 reform to the General Health Law, which prohibits cosmetic animal testing for cosmetic ingredients, finished cosmetic products, and their mixtures, restricts the manufacture, import, and marketing of animal-tested cosmetics, and establishes a transition toward alternative methods [270]. Taken together, these developments suggest a gradual trend toward greater coordination in NAM-related regulatory approaches across North America.

### South America

In Brazil, the regulatory adoption of NAMs is primarily supported by Federal Law No. 11,794/2008 (Arouca Law), which established the National Council for the Control of Animal Experimentation (CONCEA) and formally incorporated the principles of the 3Rs into national legislation governing animal experimentation. Within this framework, CONCEA is responsible for recognizing validated alternative methods and may establish mandatory timelines for their adoption once equivalent methods are available [214, 215].

Several institutional initiatives support the development, validation, and dissemination of NAMs. The Brazilian National Network for Alternative Methods (RENAMA), originally created in 2012 and relaunched in 2021, functions as a collaborative network of laboratories that develop, test, and conduct interlaboratory studies to generate reliable experimental data [214, 216]. The Brazilian Center for the Validation of Alternative Methods (BraCVAM), officially launched in 2013, coordinates the scientific evaluation and validation of these candidate methods, providing technical assessments that underpin regulatory recognition by CONCEA [271]. Many of these activities follow guidelines established by the OECD, promoting international harmonization and global acceptance of generated data [192, 271].

Once methods are recognized by CONCEA, sector-specific regulatory agencies, primarily the Brazilian Health Regulatory Agency (ANVISA), the Brazilian Ministry of Agriculture and Livestock (MAPA), and the Brazilian Institute of Environment and Renewable Natural Resources (IBAMA), incorporate them into regulatory frameworks for human health products, cosmetics, foods, chemicals, and environmental risk assessment [214]. Although several in vitro methods are already in use, particularly in cosmetic safety and toxicological testing, the regulatory pathway for advanced platforms such as MPS remains under development, reflecting ongoing challenges in methodological standardization, validation frameworks, and guidance for next-generation NAM technologies.

At the regional level, the Mercosur Platform for Alternative Methods (PReMASUL), within the framework of the Common Market, plays a strategic role in advancing NAM implementation across South America. The platform promotes capacity building through training programs, technical workshops, and collaborative initiatives involving countries such as Brazil, Argentina, Paraguay, and Uruguay, as well as associated partners including Chile and Colombia [214, 217, 218]. In addition, PReMASUL strengthens regional and international cooperation, including with European partners, by supporting knowledge exchange and enhancing laboratory infrastructure for the implementation of alternative methods. These efforts contribute to reducing disparities in technical capacity and to advancing the adoption of NAMs across the region [214, 218].

### Asia–Pacific

Historically viewed as fast followers in alternative testing, regulatory bodies across the Asia–Pacific region are rapidly evolving their policies, spurred by technological progress in bioengineering and sweeping shifts in consumer market regulations.

#### South Korea

South Korea has increased its engagement in NAMs through strategic government investments and regulatory restructuring. The Ministry of Food and Drug Safety (MFDS) established the Korean Center for the Validation of Alternative Methods (KoCVAM) in 2009 to oversee the evaluation and validation of alternative test methods developed in Korea and to support their international harmonization and contribution to OECD Test Guidelines [220]. As part of these efforts, Korea has contributed to the international recognition of human tissue models, including MCTT HCE™ under OECD TG 492 and KeraSkin™ [220–222, 236]

, which was validated as a similar reconstructed human epidermis method under OECD TG 439 performance standards. More recently, South Korea’s NAM agenda has evolved beyond isolated institutional efforts into a broader national strategy, reflecting growing policy recognition that alternative methods are needed not only to address animal welfare concerns, but also to improve the scientific relevance and predictive value of safety assessment. Legislatively, South Korea’s commitment is reflected in the introduction of the Promotion of Alternative Animal Methods (PAAM) Act in 2020 and the Vitalization of Alternative Animal Methods (VAAM) Act in 2022, which aim to promote the development of state-of-the-art non-animal methods and establish 5-year national master plans. Under the "Regulatory Science Innovation Roadmap," the government designated organoids and organ-on-chip platforms as "innovative strategic technologies," which was accompanied by expanded national R&D funding. This policy direction is being implemented through an increasingly coordinated inter-ministerial framework: MFDS leads method validation and regulatory standardization, while the Ministry of Climate, Energy and Environment (MCEE) is supporting longer-term adoption in chemical safety assessment, including a roadmap to replace more than 60% of toxicity data required for chemical registration with alternative methods by 2030 [272].

To solidify its infrastructure, the MCEE led an approximately KRW 33.4 billion investment to construct the nation’s first dedicated animal-free testing facility in Incheon, which is slated for completion in 2026. This facility will serve as an evaluation and certification hub for 3D tissue models and computer-based prediction systems. Korea was also among the early adopters in the cosmetics sector, banning the sale of animal-tested cosmetics in 2016. These governmental initiatives are further strengthened by collaboration with academia and research institutes, which are actively developing next-generation NAM platforms and supporting workforce training in non-animal methodologies. Taken together, South Korea’s approach has moved beyond ethical advocacy alone and is increasingly framed as a science-driven regulatory innovation strategy, combining legislative support, validation capacity, and advanced biotechnology infrastructure.

#### Japan

Japan’s regulatory framework takes a highly coordinated, science-centric approach. The Pharmaceuticals and Medical Devices Agency (PMDA) [224] actively champions the integration of NAMs not merely to address ethical concerns, but to significantly improve the predictive accuracy of safety evaluations through human-relevant systems. Working in tandem with the Japanese Center for Validation of Alternative Methods (JaCVAM) [225], Japan actively participates in international consortia, including the ICH, to ensure that NAM validation standards are harmonized across borders.

#### China, India, Singapore, Taiwan, and Thailand

In broader Asia, sweeping policy shifts in consumer product testing highlight a regional ethical and regulatory transition [273]. For decades, China required mandatory animal testing for imported cosmetics, functioning as a major hurdle for cruelty-free brands. However, following the implementation of the Cosmetics Supervision and Administration Regulation (CSAR) in 2021, China’s National Medical Products Administration (NMPA) introduced a regulatory framework under which imported “ordinary” (non-special-use) cosmetics may be exempted from mandatory animal testing under specified conditions, permitting companies to submit NAM-based safety assessments. Although some post-market surveillance testing remains, this shift effectively spared hundreds of thousands of animals and signaled China’s growing openness to in vitro safety science. India established regional precedence earlier by instituting a complete ban on animal testing for cosmetics in 2014. Furthermore, regulatory authorities across Singapore, Taiwan, and Thailand are increasingly forming partnerships with international NAM consortia to evaluate the applicability of MPS in biomedical research and drug evaluation.

#### Australia and New Zealand

Australia and New Zealand are gradually advancing the integration of NAMs, primarily through regulatory alignment with international frameworks and increasing participation in global validation efforts. In Australia, the National Industrial Chemicals Notification and Assessment Scheme (NICNAS), now restructured under the Australian Industrial Chemicals Introduction Scheme (AICIS) [227], encourages the use of alternative methods, including read-across, in vitro data, and computational approaches, to reduce reliance on animal testing. Similarly, New Zealand’s Environmental Protection Authority (EPA NZ) [228] promotes the use of non-animal methods under the Hazardous Substances and New Organisms (HSNO) Act, particularly where scientifically justified alternatives are available.

Both countries benefit from strong alignment with OECD TGs and the MAD system, ensuring that NAM-based data are internationally recognized [193]. While neither country has implemented comprehensive legislative bans comparable to the EU cosmetics regulation, their regulatory frameworks are increasingly supportive of NAM adoption, particularly in chemical safety assessment and environmental risk evaluation. Continued engagement with OECD initiatives and international collaborations is expected to further accelerate the uptake of human-relevant, non-animal methodologies in the region.

### Africa

The regulatory landscape for NAMs in Africa is characterized by emerging regional harmonization efforts, progressive capacity-building activities, and increasing alignment with international standards. South Africa provides an important regional example through its participation as a full adherent to the OECD MAD system, which supports the international acceptance of nonclinical safety data generated under OECD Test Guidelines and GLP principles [274, 275].

Beyond national leadership, pan-African regulatory convergence is increasingly supported by African Union initiatives. The African Medicines Regulatory Harmonization (AMRH) initiative has promoted regional joint review procedures, reliance mechanisms, and alignment of national regulatory systems. The African Medicines Agency (AMA), whose treaty entered into force in 2021 and had been ratified by 32 Member States as of May 2026, is now being operationalized as a continental mechanism for strengthening regulatory coordination and harmonization [275–277]. Although AMA is not specifically focused on NAMs, its alignment with international regulatory standards may facilitate the future integration of NAM-derived evidence into pharmaceutical safety assessment.

Nevertheless, broader NAM implementation in Africa remains constrained by limited technical infrastructure, uneven access to GLP-certified laboratories, restricted availability of advanced in vitro and in silico platforms, and gaps in specialized toxicology training [274, 278]. International collaborations and capacity-building initiatives, including activities led by the Animal-Free Safety Assessment (AFSA) Collaboration, may help build regulatory confidence and support the gradual uptake of NAM-based approaches in cosmetics, chemicals, and consumer product safety assessment [279].

Across regions, the regulatory trajectory for NAMs in nanotechnology therefore depends on convergence between two forms of standardization: context-of-use-based validation of the biological or computational method and harmonization of nanomaterial-specific information, including nanoform identity, dispersion state, exposure scenario, delivered dose, material transformation, and data integrity. This dual requirement distinguishes regulatory acceptance of NAMs for nanomaterials from the broader adoption of NAMs for conventional chemicals and provides the basis for the industrial implementation issues discussed in Sect. 4.

## Industrial applications and implementation

**Fig. 4:**
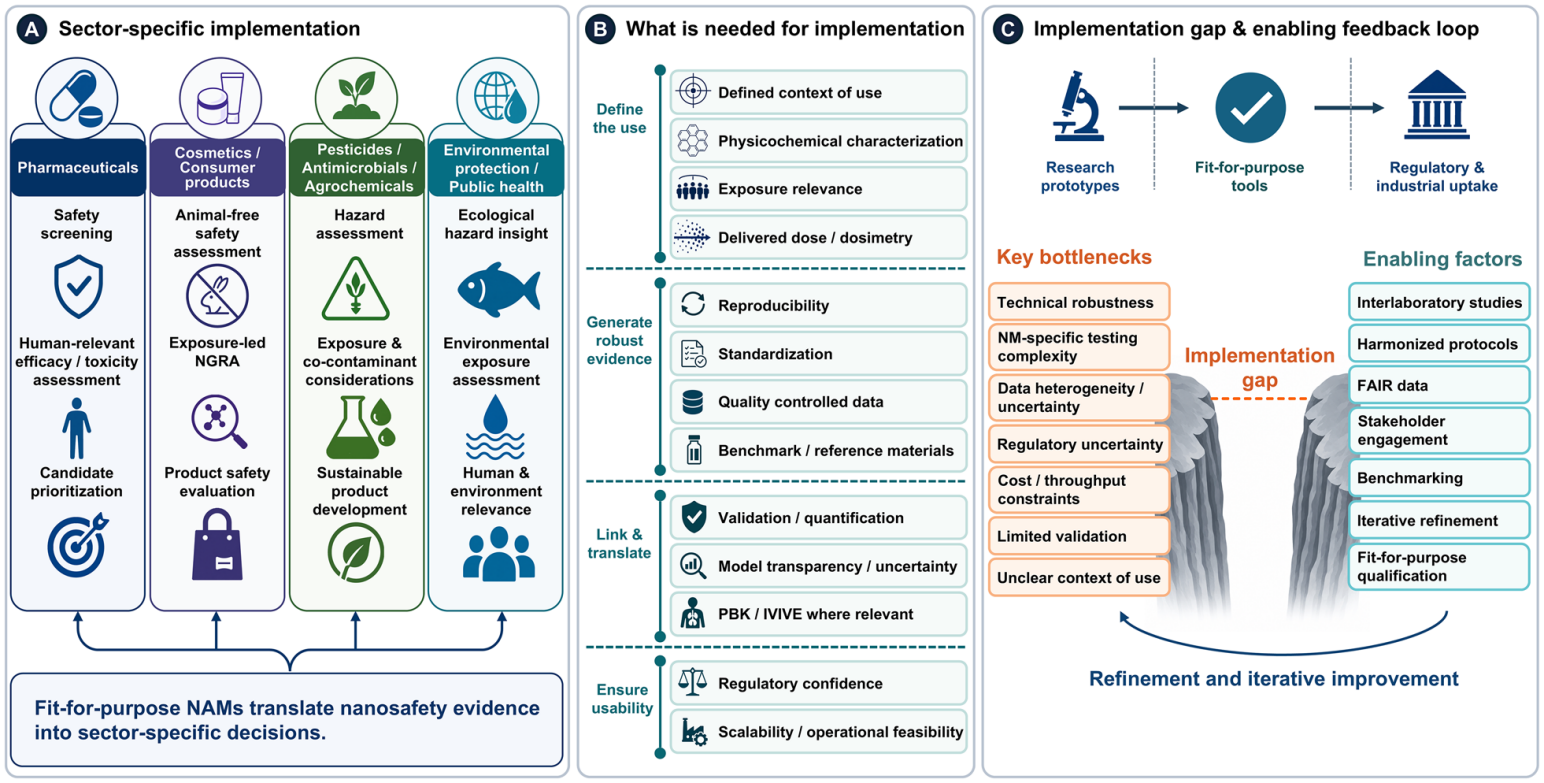
Industrial implementation and translational bottlenecks of NAMs in nanotechnology

The transition of NAMs from academic proof-of-concept to industrial implementation is a critical step toward making human-relevant and target species–relevant safety assessment usable in product development and regulatory decision-making. Driven by ethical expectations, legislative reforms, and the limited translational predictivity of conventional animal models, NAMs are increasingly being adopted for screening, de-risking, candidate prioritization, and regulatory support, although implementation maturity differs across pharmaceuticals, cosmetics and consumer products, agrochemicals, antimicrobials, and environmental health applications. In nanotechnology, this transition is especially important because nanomaterials, nanomedicines, nanopesticides, nano-enabled products, and micro- and nanoplastics require fit-for-purpose approaches that account for material-specific properties, exposure conditions, and biological responses across human and environmental systems. This section examines how NAMs are being implemented across these contexts, while highlighting recurring barriers such as validation, scalability, quality control, data integration, and regulatory confidence. In line with implementation-focused NGRA practice, these sector examples are evaluated not only as instances of NAM adoption, but as decision workflows in which context of use, exposure scenario, dose definition, material transformation, uncertainty, and evidence integration determine whether NAM-derived data can support nanosafety decisions. Figure 4 organizes these sector-specific implementation pathways by linking application areas to fit-for-purpose requirements and to bottlenecks and feedback loops that shape movement from research prototypes toward regulatory and industrial uptake.

### Pharmaceutical industry

The pharmaceutical industry is increasingly deploying NAMs to address the profound inefficiencies of the traditional drug development pipeline, where approximately 90% of clinical drug candidates fail despite encouraging preclinical evidence. This paradigm shift was formally catalyzed by the FDA Modernization Act 2.0 in 2022, which removed the explicit animal-testing requirement for new drug development while preserving regulatory discretion, and was further reinforced by the FDA’s 2025 roadmap to reduce animal testing and its subsequent draft guidance on the use of NAMs in drug development [256–258].

To leverage this regulatory flexibility, pharmaceutical companies are increasingly investing in and evaluating MPS, including organ-on-chip platforms [26, 280]. These models can recapitulate selected human physiological processes, including transport, biodistribution-related barriers, and tissue–tissue interactions, in ways that may improve translational relevance compared with animal proxies [26]. A landmark achievement in this space was the formal acceptance of Emulate’s Liver-Chip into the FDA’s ISTAND pilot program after it successfully predicted drug-induced liver injury (DILI) with 87% sensitivity, detecting hepatotoxic drugs that had previously passed animal testing [263]. Industry-reported case studies also suggest emerging regulatory openness to human-relevant efficacy models; for example, Qureator has reported that SillaJen’s BAL0891 combination-therapy investigational new drug application was supported in part by efficacy data generated using its vascularized tumor–immune microenvironment platform [281, 282].

NAMs in nanomedicine and nanotechnology contexts, particularly organ-on-chip platforms, are becoming important for evaluating the safety and performance of nanomaterials and nanomedicine formulations [283]. These systems can better capture human-relevant barrier interactions, cellular uptake, transport- and distribution-related processes, and cytotoxic responses than conventional static in vitro models, and may therefore help bridge the long-recognized gap between extensive preclinical NM research and its still limited rate of clinical translation.

For nanomedicine applications, however, industrial translation depends on exposure fidelity within the device as much as on biological complexity. Particle adsorption or deposition within PDMS devices, tubing, membranes, or other chip materials can alter the administered-to-delivered dose relationship, while formulation-dependent aggregation or dissolution may change uptake, barrier transport, and apparent cytotoxicity. Qualification of MPS for nanomedicine and nano-enabled products therefore requires verification of device-material compatibility, particle recovery, dispersion stability, and exposure profiles under flow.

Furthermore, the industry is increasingly shifting away from using non-human primates (NHPs) for the safety testing of monoclonal antibodies (mAbs) [257]. Because animal studies may fail to predict human-specific cytokine release and immunotoxicity, developers are increasingly using in vitro human cytokine release assays and immune-integrated organ-chip models to complement, reduce, or potentially replace selected primate studies [260, 261, 280].

Simultaneously, AI and in silico modeling are increasingly used to support hit-to-lead optimization and early de-risking, but their industrial value depends on data quality, model transparency, applicability-domain definition, and expert oversight [284, 285]. AI-driven platforms, including generative-AI-supported candidate design workflows, illustrate how in silico approaches can support candidate prioritization and progression into testing, provided that model outputs remain linked to interpretable biological and safety-relevant evidence [285]. Companies such as PharmCADD are deploying quantum computing-integrated AI (Pharmulator™) to help identify key safety liabilities, including cardiotoxicity and DILI, during early drug design [286, 287]. To accelerate validation and regulatory readiness, major pharmaceutical companies are collaborating through cross-industry and public–private initiatives, such as the IQ MPS Affiliate and the FNIH-led NAMs Validation and Qualification Network (VQN), to support qualification, data sharing, and context of use alignment [280, 288].

### Cosmetics and consumer products

The cosmetics and personal care sector represents one of the most advanced areas of NAM implementation, with the primary objective of enabling human-relevant safety assessment. This transition was catalyzed by legislative mandates, particularly the 7th Amendment to the EU Cosmetics Directive and Regulation (EC) No 1223/2009, which established testing and marketing bans for cosmetics developed using animal testing and thereby accelerated the adoption of non-animal approaches [250, 289]. Consequently, the sector has increasingly moved toward NGRA frameworks, which use NAM-based, non-animal evidence to support human safety decisions [290]. In the cosmetics context, NGRA is commonly defined as an exposure-led, hypothesis-driven risk assessment approach that integrates NAMs to support human-relevant safety decisions without reliance on animal data [291].

NAMs in this context encompass in vitro, in chemico, in silico, and read-across methodologies, often combined within DAs and IATA. Their regulatory acceptance has been supported by structured validation principles, including those outlined in OECD Guidance Document 34 for the validation and international acceptance of new or updated test methods [192]. As a result, NAMs for local toxicity endpoints are well-established, including reconstructed human epidermis models for skin irritation [236], 3T3 neutral red uptake (NRU) phototoxicity testing [292], and DAs for skin sensitisation [23]. For skin sensitisation, these methods are typically interpreted within AOP frameworks to enhance mechanistic relevance [293].

A defining feature of industrial implementation is the integration of NAMs into exposure-led, hypothesis-driven NGRA workflows. Regulatory integration of NAMs continues to expand. The Scientific Committee on Consumer Safety (SCCS), through its 12th revision of the Notes of Guidance, has formally incorporated NAM-based approaches, including PBK modeling and NGRA principles, into cosmetic safety evaluations [251]. For example, local effects are evaluated using validated NAMs, while systemic toxicity can be addressed through threshold-of-toxicological-concern approaches for low-exposure scenarios, in silico tools such as QSAR models (e.g., OECD QSAR Toolbox, VEGA), read-across approaches, or PBK modeling using in silico and in vitro inputs [251]. These assessments can be further strengthened by IVIVE and PBK modeling to derive human-relevant exposure thresholds [294]. Case studies (e.g., coumarin) demonstrate the feasibility of deriving safe use levels using NAM-based NGRA frameworks [294].

Recent initiatives from the International Collaboration on Cosmetics Safety (ICCS), the US FDA, and the US EPA emphasize the transition from method development to fit-for-purpose implementation, where NAMs are evaluated based on context of use, biological relevance, and technical performance [89]. The key lesson from this sector is that implementation is driven less by the availability of individual assays than by transparent, reproducible decision workflows that link exposure estimates, bioactivity points of departure, PBK/IVIVE modeling, and weight-of-evidence interpretation to a defined safety question. While regulatory acceptance continues to evolve, a central question remains whether NAMs can provide a level of confidence comparable to traditional in vivo methodologies for regulatory decision-making [290].

Despite these advancements, significant challenges remain, particularly for the systemic toxicity endpoints, including repeated-dose toxicity, reproductive toxicity, and complex endocrine effects, where fully validated NAMs are still lacking [251, 290]. Nonetheless, through collaborative initiatives and integrated approaches, the cosmetics sector provides a mature, scalable model for NAM-based safety assessment.

### Pesticides and antimicrobials

The pesticide and antimicrobial sectors—including plant protection products, biocides, and nanotechnology-based antimicrobial applications—represent complex arenas for NAM integration because of their extensive regulatory data requirements, diverse exposure scenarios, and increasing use of nanotechnology to enhance active ingredient efficacy and delivery. The conventional safety assessment paradigm for agrochemicals has historically relied heavily on standardized animal studies, including the rodent cancer bioassay [295], which, despite decades of use, presents well-documented limitations in translational predictability and remains resource- and time-intensive [296]. A paradigm shift is now underway, driven by a convergence of scientific advancement, regulatory openness, and ethical imperatives.

Nanopesticide formulations include nanoemulsions, polymeric nanocapsules, and inorganic engineered nanomaterials such as metals, metal oxides, and nanoclays, which may enable controlled release, improved foliar adhesion, or targeted delivery of active ingredients, potentially reducing the amount of active substance required per application [191, 297]. However, the same properties that confer agronomic advantages—altered environmental fate, modified dissolution kinetics, and differential uptake by non-target organisms—also raise fundamental questions about whether existing environmental risk assessment frameworks, originally developed for conventional chemical formulations, are fit for purpose for nanopesticides. Existing test methods for environmental fate, ecotoxicity, and exposure assessment may therefore require systematic adaptation to account for nanomaterial-specific phenomena, including particle agglomeration, surface corona formation, altered bioavailability, and size-dependent biological uptake [297]. Thus, for nanopesticides and nano-enabled antimicrobials, Eco-NAM implementation depends not only on replacing an animal endpoint, but on representing the nanoform encountered by non-target organisms under realistic dispersion, transformation, bioavailability, and exposure conditions. To address this, the industry is transitioning toward knowledge-based, tiered assessment models that prioritize in silico toxicokinetic modeling and in vitro assays. Regulatory frameworks for conventional pesticides, including Regulation (EC) No 1107/2009 in the EU and the Federal Insecticide, Fungicide, and Rodenticide Act (FIFRA) in the US, impose comprehensive data requirements that have traditionally relied substantially on animal-based testing [298, 299]. A major structural barrier to NAMs integration in this sector is that the documentation underpinning these requirements has not been systematically updated to reflect modern testing methodologies, creating institutional inertia that slows the uptake of scientifically validated alternatives even where regulatory flexibility formally exists [300]. In response, forward-looking conceptual models for agrochemical safety evaluation have prioritized exposure-driven, tiered, and adaptable assessment frameworks that integrate high-throughput in vitro assays, in silico toxicokinetic modeling, and NAM-derived mechanistic data, while emphasizing transparency, collaboration, open data sharing, and global harmonization of acceptance criteria [301]. Moreover, the European Food Safety Authority (EFSA) has substantially supported the development, evaluation, and application of NAMs for DNT, including their use in EFSA-driven case studies for AOP-based IATAs and PBK/QIVIVE-supported interpretation of DNT IVB data for pesticide active substances [302–304].

The intersection of nanotechnology with antimicrobial applications further complicates this landscape. Nanotechnology-based antimicrobials—including silver, zinc oxide, and copper NPs—are increasingly being explored as alternatives to conventional biocides in agricultural settings, partly in response to growing resistance among plant pathogens to existing chemical classes. In parallel, per- and polyfluoroalkyl substances (PFAS) have been reported in pesticide products as active ingredients or degradates, inert ingredients, or contaminants from fluorinated plastic containers [305]. Their presence may introduce persistent contaminants with documented toxicological concerns into pesticide exposure scenarios. Integrating exposure assessment for such co-contaminants into pesticide NGRA frameworks, and developing or qualifying NAMs capable of detecting PFAS-relevant modes of action at environmentally relevant concentrations, therefore represents an emerging priority for pesticide risk assessment and nano-enabled formulation evaluation.

### Environmental protection and public health

Traditional ecological risk assessment (ERA) relies heavily on apical in vivo data from standardized test species, such as fish, birds, and invertebrates, to derive environmental quality standards [306]. More broadly, the application of NAMs to environmental protection and public health represents one of the most compelling yet underexplored frontiers in regulatory science, as conventional ERA frameworks remain strongly dependent on animal-intensive apical endpoints and are increasingly recognized as insufficiently reflective of environmentally realistic exposure complexity [306].

In the context of nanotechnology, these limitations are further amplified [191]. When released into aquatic, terrestrial, and atmospheric compartments, NMs undergo complex transformations—including agglomeration, dissolution, surface corona formation, and biotransformation—that fundamentally alter their ecotoxicological behavior relative to their pristine physicochemical characterization, thereby challenging conventional ERA frameworks originally developed for soluble chemicals [191].

To overcome these hurdles, regulatory science is turning to advanced NAM ecosystems [306]. For environmental nanosafety, the central implementation challenge is to make these tools operate as an integrated, exposure-aware evidence system rather than as isolated assays. Such systems need to connect material transformation, target-species relevance, bioavailability, dose metrics, and cross-species extrapolation to the protection goal being addressed, thereby enabling predictive ecological risk evaluation that accounts for the dynamic behavior of nanoforms in complex environmental matrices. To address the challenge of limited toxicity data across diverse wildlife, ecotoxicologists are increasingly using bioinformatics tools and cross-species extrapolation approaches, such as SeqAPASS and G2P-SCAN, to predict whether toxicological pathways conserved in humans or rodents are applicable to thousands of non-model environmental species [307, 308]. Regulatory frameworks are also employing rule-based IATA, such as Canada’s Ecological Risk Classification (ERC2), to prioritize substances based on combined hazard and exposure metrics [12, 20, 21, 246, 309].

In the realm of nanotoxicity and public health, researchers are deploying high-throughput optical imaging combined with Fourier-based frequency analysis to monitor the cardiac dynamics of *Daphnia magna* at scale [117]. This enables environmental toxicologists to capture the stochastic nature of NP interactions and move from mean-based observational values toward statistically robust, distributional analyses. Furthermore, the European Research Cluster to Understand the Health Impacts of Micro- and Nanoplastics (CUSP) is applying NAMs to develop human-health risk assessment frameworks for micro- and nanoplastics (MNPs) [310]. Its activities also support improved particle detection methods and generate evidence intended to inform European regulatory discussions, including those relevant to REACH.

## Future perspectives, current challenges, and concluding remarks

### Toward “Smart NAMs” and digital twins

Recent advances in bioengineering, embedded sensing, automation, and computational modeling are moving NAMs beyond static assay systems toward so-called “Smart NAMs”: adaptive, sensor-integrated platforms that combine advanced in vitro models with continuous monitoring and data-driven interpretation. In these systems, biological models such as organoids, barrier cultures, and MPS are coupled with embedded sensors, automated data acquisition, and machine learning–based analysis to generate longitudinal datasets on dynamic biological responses [26, 140, 141, 280, 284]. This shift from isolated endpoint measurements to time-resolved, multidimensional response trajectories can improve the mechanistic resolution of NAM platforms and help distinguish transient adaptive responses from persistent perturbations that may be more relevant to hazard assessment.

A closely related concept is that of digital twins, computational replicas of biological systems that integrate experimental data with physiological modeling to simulate tissue- or organism-level responses to chemical or material exposure [151, 171, 311]. By linking experimental NAM outputs with computational frameworks such as PBK models and AI-driven predictive tools, digital twins can support the simulation of exposure scenarios, dose–response relationships, and long-term biological outcomes. Such hybrid experimental–computational ecosystems could enable closed-loop toxicology workflows, in which experimental results refine computational models and model predictions guide targeted follow-up testing. Bridging the persistent gap between academic model development and industrial implementation will require academic developers to design NAMs with predefined contexts of use and transferability in mind, industry to evaluate scalability and quality control requirements, and regulatory and standardization bodies to define performance, reproducibility, and reporting expectations for routine SSbD application [89, 284, 312, 313].

### Safe and sustainable by design (SSbD)

SSbD is defined as an approach that supports the design, development, production, and use of chemicals and materials while providing a desirable function or service and avoiding or minimizing harmful impacts on human health and the environment throughout their life cycle [248]. Originally proposed by the European Commission’s JRC in 2022 in support of the EU Chemicals Strategy for Sustainability and the broader European Green Deal, the SSbD framework was revised in 2025 to address limitations identified through stakeholder testing across multiple industrial and regulatory contexts [248, 312, 314, 315]. SSbD builds on earlier safe-by-design efforts focused on nanomaterials, while expanding the framework to integrate safety, circularity, functionality, and life-cycle sustainability into a coherent assessment process applicable across technology readiness levels [248, 316]. The consequences of SSbD for the nanotechnology and regulatory science landscape are profound. By embedding hazard, risk, and sustainability considerations early in material design and development, SSbD repositions NAMs as tools of innovation governance rather than solely as regulatory compliance instruments [191, 317]. The complementary OECD Safe(r) and Sustainable Innovation Approach (SSIA), developed for nanomaterials, advanced materials, and nano-enabled products, extends these principles beyond the EU by integrating regulatory preparedness into innovation governance, signaling growing international convergence around responsible nanotechnology development [317].

In practical terms, SSbD shifts safety assessment from a late-stage regulatory exercise to an iterative design process in which safety, functionality, circularity, and life-cycle sustainability are considered together. The rapid evolution of NAMs, ranging from advanced in vitro systems to data-driven in silico approaches, provides critical tools to operationalize this shift [39]. Increasingly sophisticated biological models, including polarized barrier cultures, organoids, and MPS, enable physiologically relevant investigation of material–tissue interactions across key exposure interfaces during early material screening. In parallel, advances in computational toxicology, machine learning, and automated data extraction are expanding the predictive capacity and scalability of hazard, exposure, and comparative material assessment. Together, these complementary approaches support the early identification of potential risks during material development and facilitate iterative design processes that integrate safety and sustainability considerations from the outset of innovation [314]. In this context, NAMs can function not only as testing tools but also as a mechanistically informed evidence engine for stage-gated SSbD workflows, supporting the comparison and prioritization of candidate materials under realistic exposure scenarios [248]. Integration with PBK/QIVIVE approaches further strengthens this role by linking bioactivity data to internal exposure and enabling more quantitative, exposure-led decision-making within NGRA frameworks [39, 151]. However, realizing the full potential of SSbD requires not only technological innovation but also coordinated action among academia, industry, regulatory bodies, standardization organizations, and metrology institutes, supported by Findable, Accessible, Interoperable, and Reusable (FAIR)-aligned data infrastructures, transparent workflows, and interoperable knowledge systems [312, 313]. Improved standardization, interlaboratory reproducibility, and regulatory alignment are therefore essential to translate emerging NAMs into robust tools for stage-gated SSbD decision-making. Bridging the persistent gap between academic model development and industrial implementation will be a key step toward enabling the routine use of NAMs-driven SSbD frameworks for the development of safer and more sustainable materials and products across their life cycle [315, 318].

### Challenges and regulatory transformation for next-generation risk assessment

Despite the rapid progress of NAMs, several scientific, technical, and regulatory challenges continue to limit their broader implementation and acceptance within NGRA frameworks [9, 10, 19, 39]. One of the most significant challenges lies in the standardization, reproducibility, and transferability of advanced NAM platforms, particularly as technologies such as organoids, MPS, high-content omics platforms, and AI-driven analytical workflows evolve faster than existing validation and regulatory qualification processes [67, 89, 192, 258, 280, 288]. In nanotechnology, these challenges are further amplified by the dynamic and context-dependent behavior of nanomaterials, including agglomeration, dissolution, sedimentation, protein corona formation, and delivered-dose variability, all of which can substantially influence biological responses and interlaboratory reproducibility [37, 190, 191]. Consequently, ensuring consistent physicochemical characterization, exposure conditions, dosimetry, and data interpretation remains a critical prerequisite for regulatory confidence and industrial scalability.

At the same time, regulatory agencies face the complex task of defining which mechanistic endpoints, molecular signatures, and quantitative metrics are sufficiently robust, interpretable, and decision-relevant to support hazard identification and risk assessment. Unlike traditional toxicology, which has historically relied on observable apical outcomes in whole organisms, NAMs increasingly generate high-dimensional datasets describing pathway perturbations, cellular stress responses, transcriptomic changes, and systems-level biological adaptations [93, 94, 161–169]. Translating these mechanistic signals into regulatory decisions therefore requires clearer frameworks for uncertainty assessment, data integration, and interpretation based on context of use [10, 89, 192, 258]. In particular, approaches for evaluating biological relevance, defining applicability domains, and communicating uncertainty will be essential for broader regulatory adoption of NAM-derived evidence.

More fundamentally, the transition toward NAM-based safety assessment may require a paradigm shift in regulatory toxicology itself. Current hazard classification systems, including frameworks underlying the Globally Harmonized System (GHS) and the Classification, Labelling and Packaging (CLP) regulation, were largely developed around animal-based apical endpoints and observable adverse outcomes [319, 320]. In contrast, NAMs support a more mechanistic and pathway-oriented understanding of toxicity, anchored in MIEs, KEs, and integrated biological response networks. As a result, future regulatory frameworks may progressively evolve from reliance on overt organism-level toxicity toward the interpretation of defined molecular signatures, mechanistically anchored biomarkers, and exposure-relevant biological perturbations. Such a transition would require not only validation of new methods but also adaptation of regulatory concepts, evidentiary standards, and hazard classification paradigms.

Ultimately, the successful integration of NAMs into NGRA frameworks will depend on coordinated advances across technology development, regulatory science, data infrastructure, and international harmonization. Bridging the persistent gap between academic innovation and regulatory implementation will require stronger collaboration among academia, industry, standardization bodies, and regulatory agencies, alongside FAIR-compliant data ecosystems, transparent validation frameworks, and internationally harmonized guidance [144, 321].

### Concluding remarks

NAMs mark a shift in nanosafety assessment from retrospective, animal-centered toxicity testing toward mechanistically informed, human- and target species-relevant NGRA. This review shows that their value for nanotechnology depends on the convergence of advanced biological platforms, nanospecific analytics, in silico modeling, and robust physicochemical characterization, exposure control, and dosimetry. When applied within clearly defined contexts of use, these approaches can provide more predictive and transparent evidence for regulatory and industrial decision-making.

However, broader implementation will require harmonized standards, interlaboratory reproducibility, FAIR data infrastructures, and closer alignment among method developers, industry, and regulators. By integrating Smart NAMs, digital twins, PBK/QIVIVE, AOP-informed interpretation, and SSbD principles into iterative design and assessment workflows, the field can better translate nanotechnology innovation into safer, more sustainable, and regulatory-relevant outcomes.

## Data Availability

No new datasets were generated or analyzed during the current study. All relevant information was obtained from previously published sources, which are appropriately cited in the manuscript.

## Abbreviations

2D: Two-dimensional
3D: Three-dimensional
3Rs: Replacement, Reduction, and Refinement
AD: Applicability domain
AI: Artificial intelligence
ALI: Air–liquid interface
AO: Adverse outcome
AOP: Adverse Outcome Pathway
AutoML: Automated machine learning
BBB: Blood–brain barrier
CLP: Classification, Labelling and Packaging
CyTOF: Cytometry by time-of-flight
DA: Defined Approach
DILI: Drug-induced liver injury
DNT: Developmental neurotoxicity
DNT IVB: Developmental neurotoxicity in vitro battery
DPRA: Direct Peptide Reactivity Assay
ECM: Extracellular matrix
EDF: Enhanced dark-field imaging
EDF-HSI: Enhanced dark-field hyperspectral imaging
EFSA: European Food Safety Authority
EPA: U.S. Environmental Protection Agency
ERA: Ecological risk assessment
EU: European Union
ECVAM: European Centre for the Validation of Alternative Methods
EURL ECVAM: EU Reference Laboratory for alternatives to animal testing
FAIR: Findable, Accessible, Interoperable, and Reusable
FDA: U.S. Food and Drug Administration
GHS: Globally Harmonized System
GLP: Good Laboratory Practice
h-CLAT: Human Cell Line Activation Test
HSI: Hyperspectral imaging
HTS: High-throughput screening
IATA: Integrated Approaches to Testing and Assessment
ICH: International Council for Harmonisation
IVIVE: In vitro-to-in vivo extrapolation
KE: Key event
LLM: Large language model
LSPR: Localized surface plasmon resonance
MAD: Mutual Acceptance of Data
mAbs: Monoclonal antibodies
MIE: Molecular initiating event
ML: Machine learning
MNPs: Micro- and nanoplastics
MOA: Mechanism of action
MPS: Microphysiological systems
MWCNTs: Multi-walled carbon nanotubes
NAM: New Approach Methodology
nano-QSAR: Nano-quantitative structure–activity relationship
NGRA: Next-generation risk assessment
NIH: U.S. National Institutes of Health
NLP: Natural language processing
NM: Nanomaterial
NP: Nanoparticle
OECD: Organisation for Economic Co-operation and Development
PBK: Physiologically based kinetic
PDMS: Polydimethylsiloxane
PFAS: Per- and polyfluoroalkyl substances
QIVIVE: Quantitative in vitro-to-in vivo extrapolation
QNAR: Quantitative nanostructure–activity relationship
QSAR: Quantitative structure–activity relationship
REACH: Registration, Evaluation, Authorisation and Restriction of Chemicals
ROS: Reactive oxygen species
SCCS: Scientific Committee on Consumer Safety
scRNA-seq: Single-cell RNA sequencing
SSbD: Safe and Sustainable by Design
TEER: Transepithelial/transendothelial electrical resistance
TG: Test Guideline
TGx: Toxicogenomics
TSCA: Toxic Substances Control Act
WPMN: Working Party on Manufactured Nanomaterials
